# GADD45α drives brown adipose tissue formation through upregulating PPARγ in mice

**DOI:** 10.1038/s41419-020-02802-5

**Published:** 2020-07-27

**Authors:** Wenjing You, Ziye Xu, Ye Sun, Teresa G. Valencak, Yizhen Wang, Tizhong Shan

**Affiliations:** 1 College of Animal Sciences, Zhejiang University, Hangzhou, China; 2 The Key Laboratory of Molecular Animal Nutrition, Ministry of Education, Hangzhou, China; 3 Zhejiang Provincial Laboratory of Feed and Animal Nutrition, Hangzhou, China

**Keywords:** Metabolomics, Cell proliferation, Differentiation

## Abstract

Stress can lead to obesity and metabolic dysfunction, but the underlying mechanisms are unclear. Here we identify GADD45α, a stress-inducible histone folding protein, as a potential regulator for brown adipose tissue biogenesis. Unbiased transcriptomics data indicate a positive correlation between adipose *Gadd45a* mRNA level and obesity. At the cellular level, *Gadd45a* knockdown promoted proliferation and lipolysis of brown adipocytes, while *Gadd45a* overexpression had the opposite effects. Consistently, using a knockout (*Gadd45a*^*−/−*^) mouse line, we found that GADD45α deficiency inhibited lipid accumulation and promoted expression of thermogenic genes in brown adipocytes, leading to improvements in insulin sensitivity, glucose uptake, energy expenditure. At the molecular level, GADD45α deficiency increased proliferation through upregulating expression of cell cycle related genes. GADD45α promoted brown adipogenesis via interacting with PPARγ and upregulating its transcriptional activity. Our new data suggest that GADD45α may be targeted to promote non-shivering thermogenesis and metabolism while counteracting obesity.

## Introduction

Stress can lead to metabolic dysfunction and obesity^1^. Obesity has become a global epidemic and is a major risk factor associated with several metabolic syndromes, such as type 2 diabetes, insulin resistance, heart disease, stroke, hyperglycemia, hypertension, and cancer^2,3^. Adipocytes play critical roles in systemic metabolism and energy homeostasis. In mammals, three types of adipocytes, white, brown, and beige or brite adipocytes, have been identified^4^. Among them, white adipocytes store excess energy in lipid droplets^5^, while beige and brown adipocytes burn lipids to produce heat, thus counteracting obesity^6,7^. Unlike white adipocytes, beige and classical brown adipocytes are characterized by their unique ability to transform mitochondrial energy into heat via uncoupling protein 1 (UCP1)^8,9^. In mammals therefore, non-shivering thermogenesis in BAT helps to maintain body temperature in cold environments while spending energy during times of high caloric intake^9^. Thus, better understanding of adipogenesis and its molecular regulation, especially of the brown and beige fat cells, may give rise to efficient and novel strategies for combating obesity and related metabolic disorders.

The growth arrest and DNA damage 45 (GADD45) protein family, consists of three members including GADD45α, GADD45β and GADD45γ^10,11^. GADD45α is a small (18.4 kDa) p53-regulated histone-fold protein known to be induced by varieties of genotoxic stress agents, such as hypoxia, UV radiation, ionizing radiation, oxidants, and alkylating agents^12,13^. GADD45α plays an important role in DNA repair^14^, cell cycle^15^, apoptosis^16,17^, angiogenesis^18^, senescence^19,20^, and DNA demethylation^21^. *Gadd45a*^*−/−*^ mice exhibit increased genome instability, reduced nucleotide excision repair and a higher rate of mutations^22^. Recently, Schäfer et al. found that GADD45α and ING1 (inhibitor of growth family member 1) are required for the differentiation of mouse embryonic fibroblasts^23^. The GADD45α/ING1 double-knockout mice display segmental progeria, lipodystrophy and metabolic defects^23^. During white adipogenesis, GADD45α promotes white adipocyte differentiation through epigenetic regulation^24,25^. These results suggest that GADD45α may play an important role in white adipocytes and energy metabolism. However, the role of GADD45α in brown adipocytes was unclear and the molecular mechanisms underlying the functional regulation of GADD45α in BAT remained to be determined. Furthermore, whether the expression of *Gadd45a* is correlated to obesity remains unclear.

In this study, we used unbiased transcriptomics data analysis and a GADD45α knockout (*Gadd45a*^*−/−*^) mouse model to determine the regulatory role of GADD45α in brown adipocytes and energy metabolism. We found that *Gadd45a* mRNA expression is positively correlated with fat deposition. Deficiency of GADD45α affects brown adipocytes proliferation, lipolysis, and mitochondrial biogenesis, and results in obvious metabolic phenotypes. We further revealed the molecular mechanisms underlying the roles of GADD45α in brown adipocytes. Our results demonstrate that GADD45a is a critical regulator of BAT growth and function, and suggest that may be a potential therapeutic target to combat obesity and other metabolic diseases.

## Results

### GADD45α expression is positively correlated with lipid metabolism and obesity

**Fig. 1:**
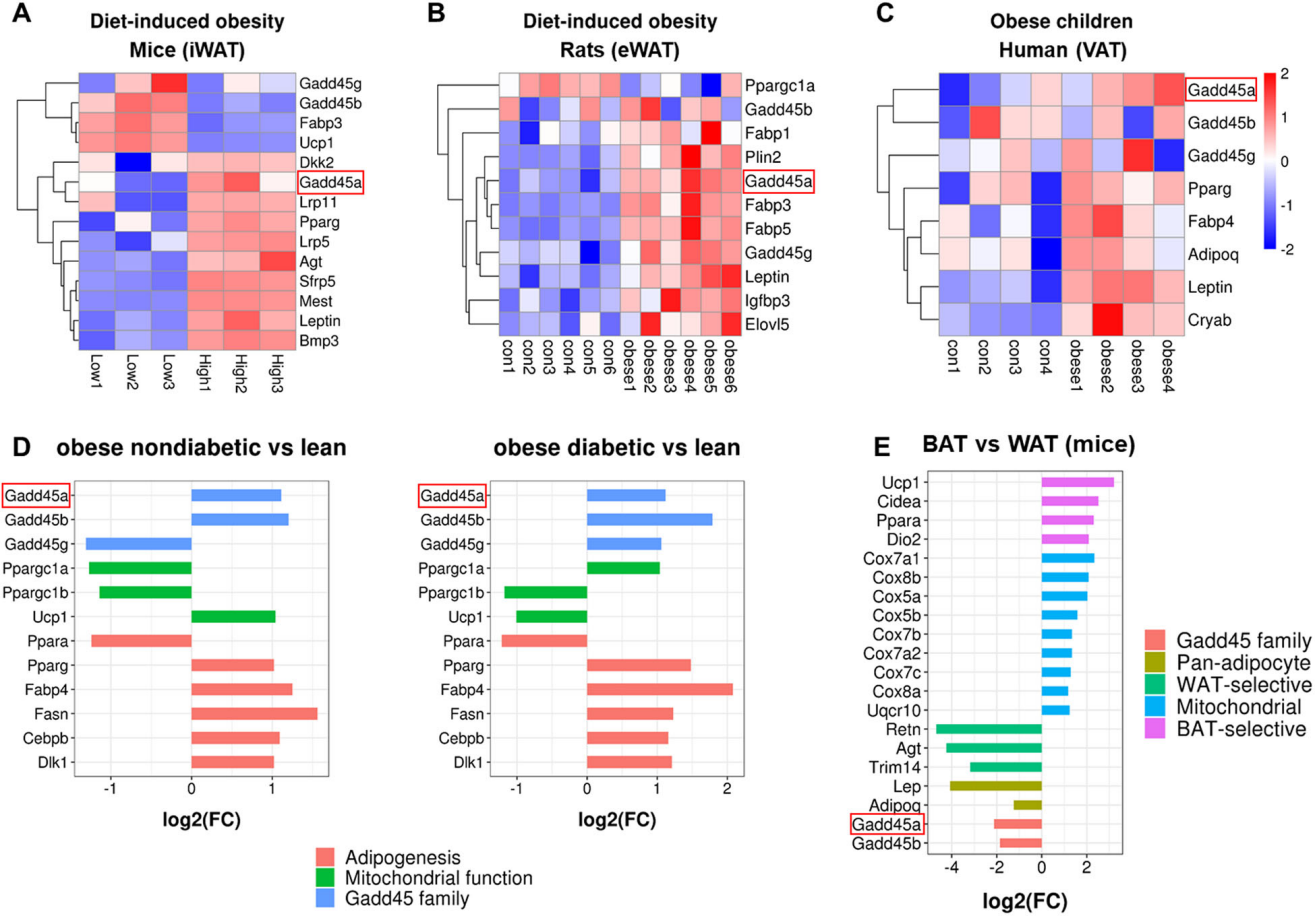
Comparison of genetic programs in diet-induced obesity and diabetes models. **a**–**c** Log2 fold changes of the lipid metabolism-related genes and *Gadd45* family in diet-induced obese mice (**a**), diet-induced obese rats (**b**), and obese children (**c**). **d** Log2 fold changes for obese nondiabetic models and obese diabetic models in human livers. **e** Log2 fold changes of the lipid metabolism-related genes, mitochondrial genes and *Gadd45* family in adipose tissues in mice.

To explore the novel genes associated with obesity, we performed a transcriptome analysis with several published datasets on adipose tissues and disease models. Notably, we found that *Gadd45a* as well as several genes related to lipid metabolism including *Lep*, were highly expressed in white adipose tissues of obese mice (Fig. 1a, GSE4692)^26,27^, rats (Fig. 1b, GSE8700)^28^ and children (Fig. 1c, GSE9624)^29^. Likewise, in human livers, high levels of *Gadd45a* were found in both the obese nondiabetic model and the obese diabetic model compared to lean groups (Fig. 1d, GSE121344). When comparing the expression of *Gadd45a* in white and brown adipocytes, we found that *Gadd45a* was highly expressed in white adipocytes in mice^27^ (Fig. 1e). These results indicate that *Gadd45a* expression is positively correlated with obesity and may represent a potential regulator of lipid metabolism and brown adipogenesis.

### GADD45α deficiency promotes brown adipocyte proliferation through upregulating cell cycle related genes

**Fig. 2:**
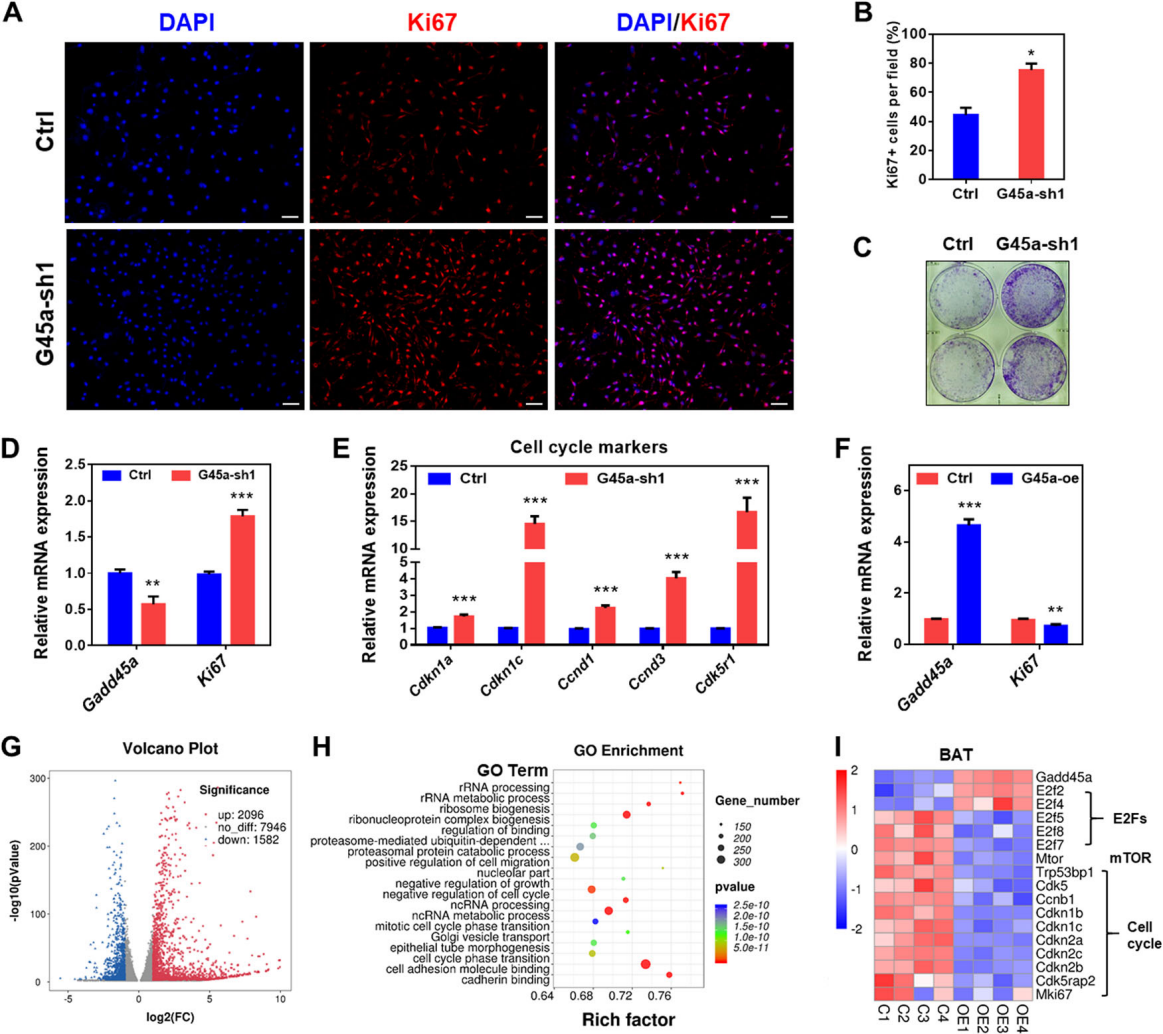
GADD45α deletion promotes proliferation of brown adipocytes. **a**, **b** Brown adipocytes stained with proliferating marker Ki67 (red) and DAPI (blue) in control and G45a-sh1 cells (**a**), and the percentage of Ki67^+^ cells (**b**). **c** Colony assay comparing growth of control and G45a-sh1 cells. **d**, **e** mRNA levels of *Ki67* and cell cycle related genes in control and G45a-sh1 cells. *n* = 4. **f** mRNA levels of *Ki67* in control and G45a-oe cells. *n* = 4. **g** Scatterplot of gene expression differences between control and G45a-oe BAT cells as determined by RNA sequencing. Shades of blue correspond to genes downregulated in the G45a-oe cells, and shades of red indicate upregulation in the G45a-oe cells. *n* = 4. |log2(Fold Change)| > 2, *p* < 0.001. **h** Gene Ontology (GO) enrichment analysis was performed with −log10 (*p* value) plotted (*x*-axis) as a function of classification meeting a *p* value of <0.001. **i** Heatmap of expressions of selected cell cycle related genes from the RNA-seq dataset. Error bars represent SEM, *P < 0.05, **P < 0.01, ***P < 0.001, two-tailed Student’s *t* test. Scale bars: 200 µm.

To study the potential role of GADD45α in regulating brown adipocyte function, we first examined whether deletion of *Gadd45a* affects brown adipocyte proliferation in culture. We designed three independent lentiviral shRNA plasmids to knockdown *Gadd45a* in brown adipocytes. Infection with the shRNA1 lentivirus led to a 70% reduction in the level of *Gadd45a*, compared to cells treated with control shRNA. Thus, shRNA1 lentivirus (G45a-sh1) was used to establish a stable *Gadd45a* knockdown cell line, which was used in the following experiments. Notably, we found a higher percentage of Ki67+ cells in the *Gadd45a* knockdown cells compared to control cells (Fig. 2a, b). Analysis of colony formation (Fig. 2c) further confirmed that GADD45α deficiency increased the proliferation and colonization of brown adipocytes. Moreover, compared to control cells, mRNA levels of *Ki67* and cell cycle markers including *Cdkn1a*, *Cdkn1c*, *Ccnd1*, *Ccnd3*, and *Cdk5r1* were significantly up-regulated in the *Gadd45a* knockdown cells (Fig. 2d, e). By contrast, *Gadd45a* overexpression inhibited brown adipocyte proliferation in vitro (Supplementary Fig. 1a–d). In addition, the mRNA level of *Ki67* was down-regulated in *Gadd45a* overexpressing (G45a-oe) cells (Fig. 2f).

To further confirm the effects of GADD45α on brown adipocyte proliferation, we applied RNA-seq to map transcriptional changes upon *Gadd45a* overexpression. We found a total of 3678 differentially expressed genes, out of which 2096 were increased and 1582 were decreased (Fig. 2g). Gene ontology (GO) enrichment analysis revealed pronounced changes in genes involved in cell cycle and growth (Fig. 2h), particularly the expressions of cell cycle related genes were downregulated (Fig. 2i) in *Gadd45a* overexpressing cells. These results suggest that deletion of *Gadd45a* promotes brown adipocyte proliferation through upregulating cell cycle-related genes.

### GADD45α deficiency inhibits brown adipocyte lipogenesis but promotes lipolysis in vitro

**Fig. 3:**
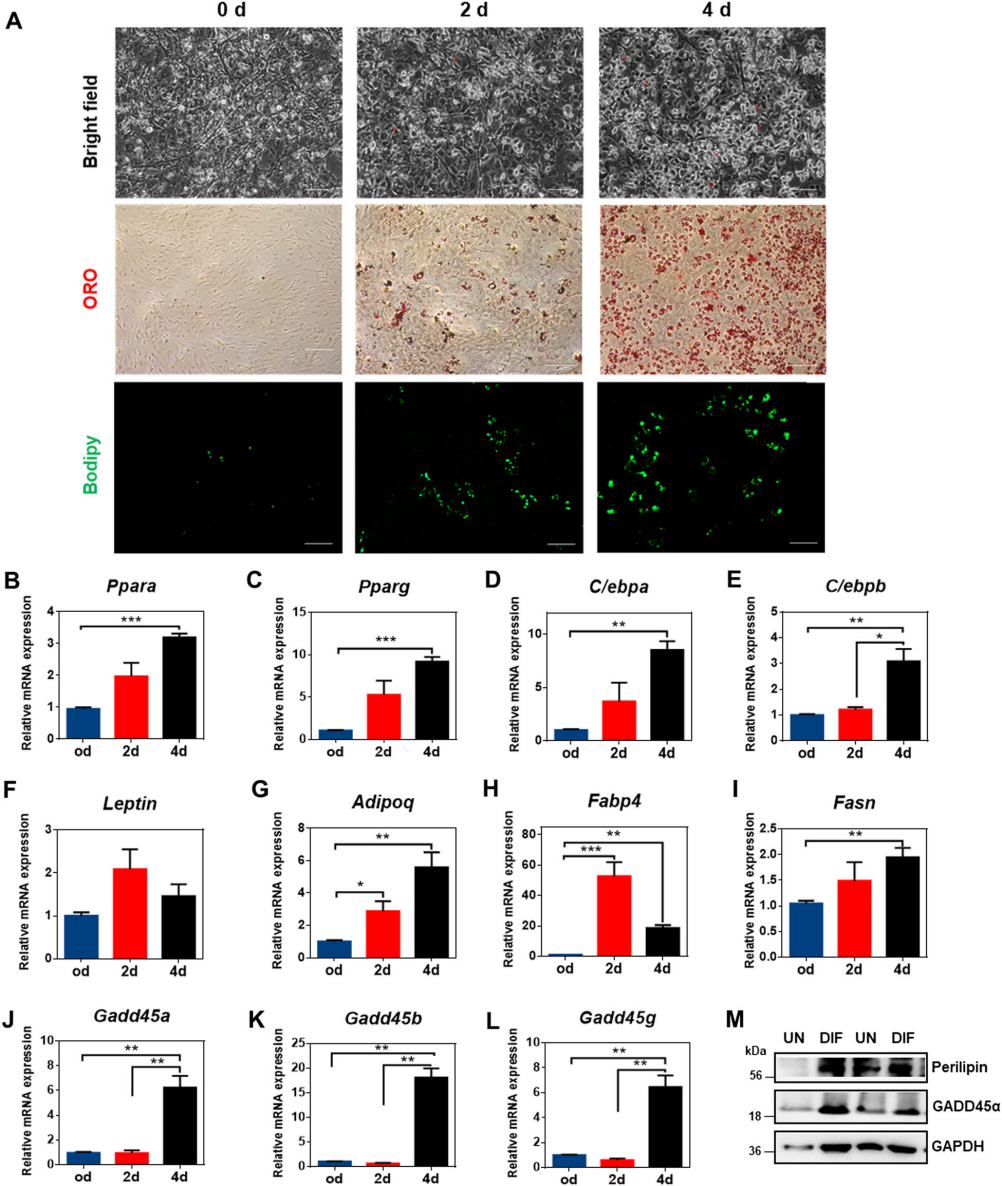
Adipogenesis of brown adipocytes at different stages. **a** Bright field and Oil Red O and bodipy staining of total lipids in differentiated brown adipocytes at different stages. Brown adipocytes were cultured in an adipogenic medium and samples were collected on 0, 2, and 4 days. **b**–**l** The relative mRNA expression of related genes (*Ppara*, *Pparg*, *C/ebpa*, *C/ebpb*, *Lep*, *Adipoq*, *Fabp4*, *Fasn*, *Gadd45a*, *Gadd45b*, and *Gadd45g*) were detected by qPCR. *n* = 4. **m** Protein levels of GADD45α and Perilipin were detected by western blot. Error bars represent SEM, *P < 0.05, **P < 0.01, ***P < 0.001, two-tailed Student’s *t* test. Scale bars: 200 µm.

To determine the role of GADD45α for the differentiation of brown adipocytes, we isolated SVF cells from BAT and examined adipogenic differentiation. Oil Red O and bodipy staining results revealed increasing lipid accumulation after adding a differentiation medium into the culture with brown preadipocytes for four days (Fig. 3a). The mRNA levels of lipogenic genes, including *Ppara*, *Pparg*, *Cebpa*, *Cebpb*, *Adipoq*, *Fabp4*, and *Fasn* were significantly upregulated (Fig. 3b–i). Consistently, mRNA and protein levels of GADD45 family proteins were also observed to be increased (Fig. 3j–m). Thus, we speculate that GADD45α may be generally related to adipogenic differentiation in brown adipocytes.

**Fig. 4:**
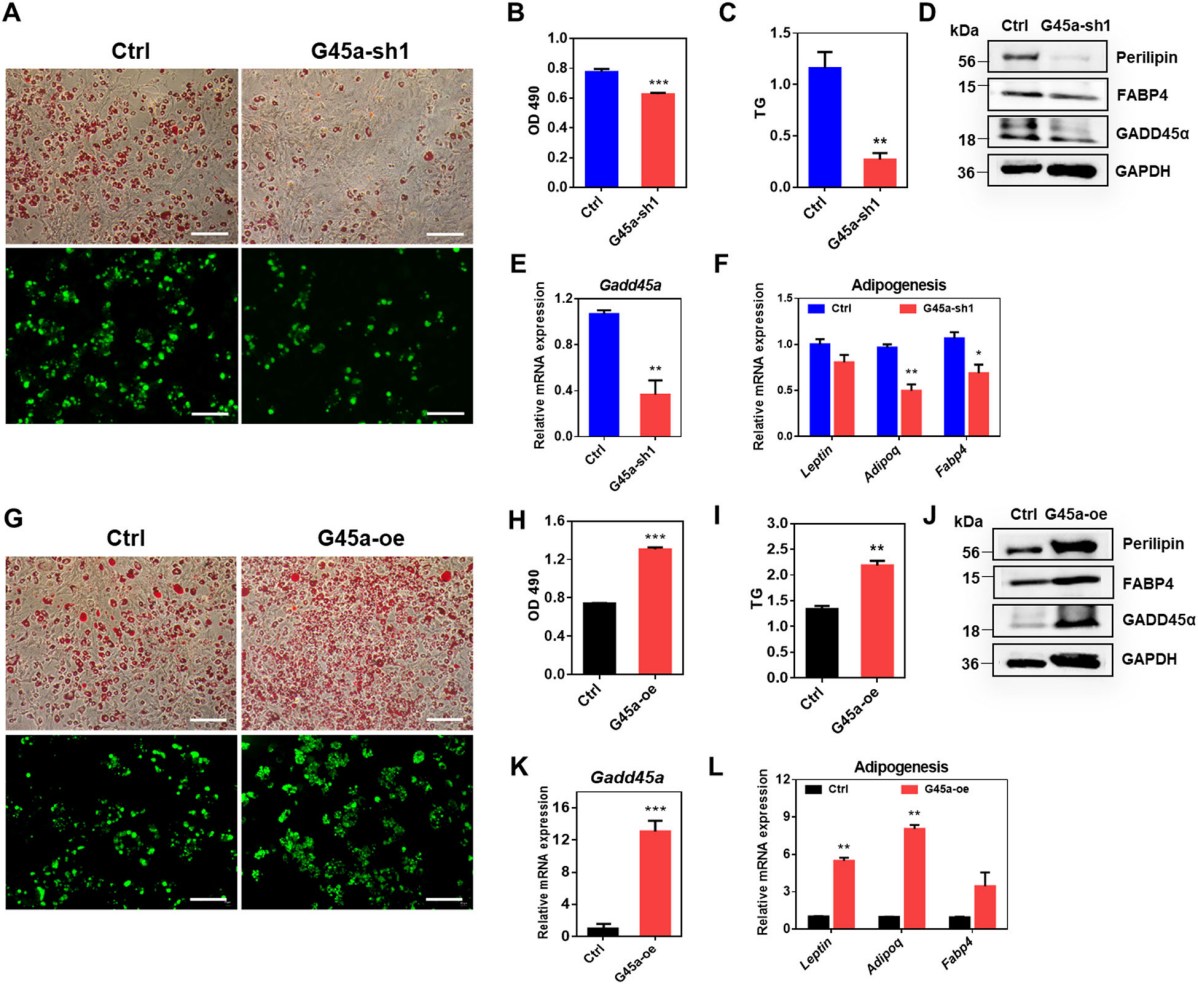
GADD45α influences adipogenic differentiation and lipid accumulation in brown adipocytes. **a** Oil Red O and bodipy staining of total lipids in differentiated brown adipocytes after treatment with control and G45a-sh1 plasmids. **b**, **c** OD 490 (**b**) and triglyceride (TG) levels (**c**) were measured. **d** Protein levels of FABP4 and Perilipin in control and G45a-sh1 cells after differentiation. **e**, **f** mRNA levels of *Gadd45a* and adipogenic related genes in control and G45a-sh1 treated cells after differentiation. *n* = 4. **g** Oil red O and bodipy staining of control and G45a-oe cells after differentiation. **h**, **i** Triglyceride (TG) levels were measured in control and G45a-oe cells based on OD490. **j** Protein levels of FABP4 and Perilipin in control and G45a-oe cells after differentiation. **k**, **l** Relative mRNA levels of *Gadd45a* and adipogenic related genes in control and G45a-oe cells after differentiation. *n* = 4. Error bars represent SEM, *P < 0.05, **P < 0.01, ***P < 0.001, two-tailed Student’s *t* test. Scale bars: 100 µm.

We further employed a loss-of-function study in cell culture. We performed adipogenic differentiation in G45a-sh1 treated BAT SVF cells. Our results revealed that *Gadd45a* knockdown robustly inhibited brown adipocyte lipogenesis and TG accumulation (Fig. 4a–c). Similarly, G45a-sh1 adipocytes expressed lower levels of perilipin protein, and lower mRNA levels of *Fabp4* and *Adipoq* than the control group (Fig. 4d–f). Lipolysis of triglycerides (TGs) ultimately results in the liberation of glycerol and free fatty acids within the fat cells^30^. We observed higher levels of glycerol release from G45a-sh1 adipocytes as compared with the controls (Supplementary Fig. 2a), suggesting G45a-sh1 indeed increased lipolysis. We also performed a gain-of-function experiment by using an adenovirus-mediated overexpression of *Gadd45a* (G45a-oe) in cultured SVF cells isolated from BAT. As a consequence, G45a-oe reduced glycerol release (Supplementary Fig. 2b) and promoted adipocyte lipogenesis by increasing mRNA expression of adipogenic related genes including *Fabp4*, *Perilipin*, *Lep*, and *Adipoq* (Fig. 4g–l). These results indicate that *Gadd45a* knockdown suppressed the process of lipogenesis but promoted lipolysis in brown adipocytes in vitro, while *Gadd45a* overexpression had the opposite effect.

### GADD45α deficiency promotes mitochondrial biogenesis in brown adipocytes

**Fig. 5:**
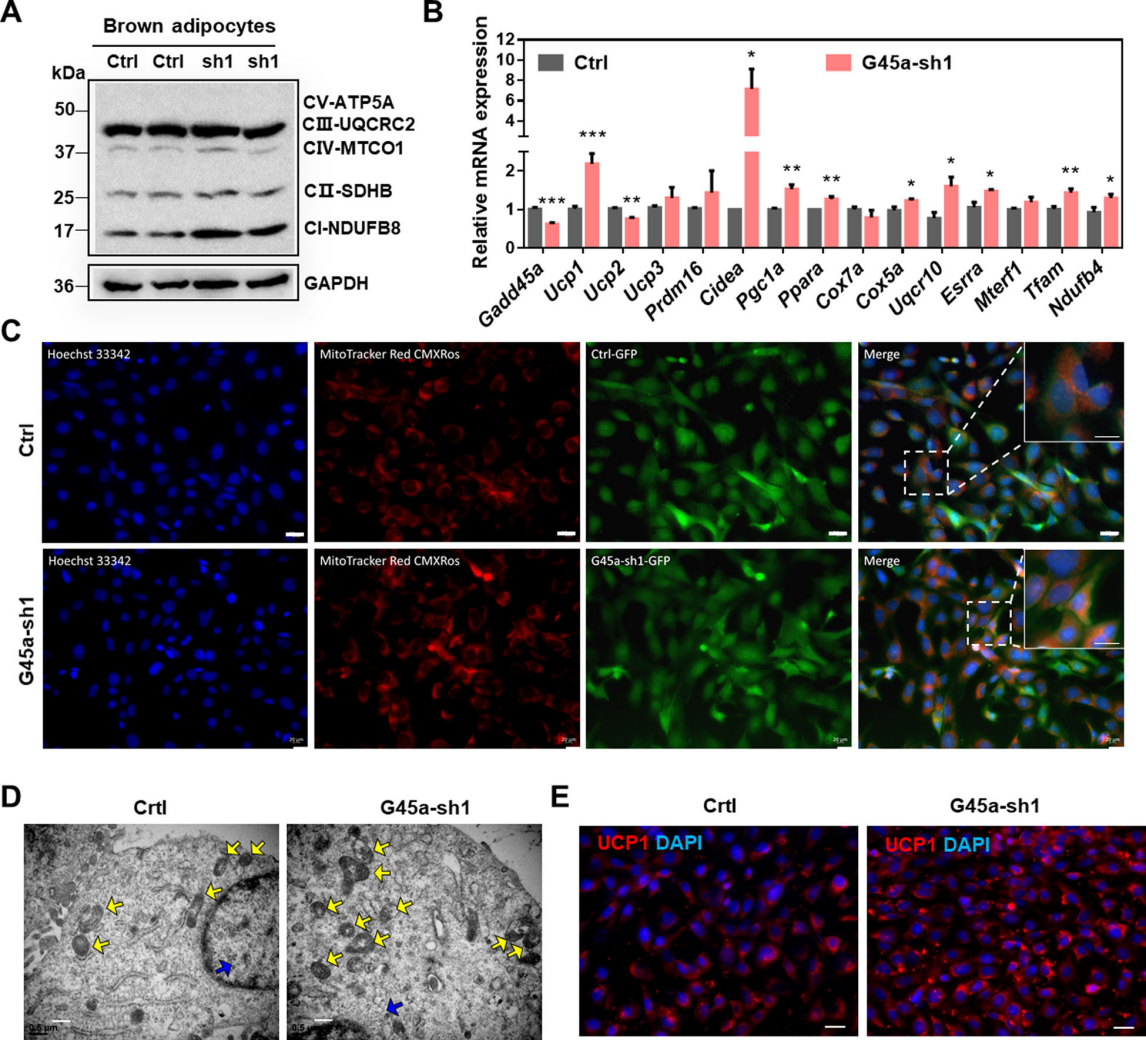
GADD45α regulates mitochondrial biogenesis of brown adipocytes. **a** The protein levels of ETC (electron transport chain) complexes (ATP5A, ATP synthase, H+ transporting, mitochondrial F1 complex, alpha 1; UQCRC2 ubiquinol-cytochrome c reductase core protein II, MTCO1 cytochrome c oxidase I, SDHB succinate dehydrogenase complex iron sulfur subunit B, NDUFB8 ubiquinone oxidoreductase subunit B8) in brown adipocytes. **b** The mRNA level of BAT-specific and mitochondrial related genes were measured by qPCR. *n* = 6. **c** The mitochondrial staining (Mito Tracker Red CMXRos) of control and G45a-sh1 BAT cells. Scale bar, 50 µm. **d** The structure of each mitochondrion was detected by transmission electron microscope (TEM) in control and G45a-sh1 cells. Scale bar, 0.5 µm. **e** UCP1 staining in control and G45a-sh1 treated BAT cells. Scale bar, 50 µm. Error bars represent SEM, *P < 0.05, **P < 0.01, ***P < 0.001, two-tailed Student’s *t* test.

To examine whether GADD45α deficiency affects mitochondrial biogenesis, we studied the expression of genes relating to mitochondrial biogenesis in vitro. We found that the protein levels of complex CI (NDUFB) and C II (SDHB) were increased in G45a-sh1 brown adipocytes, relative to controls (Fig. 5a). Moreover, BAT-specific (*Ucp*1, *Cidea*, *Pgc1a*, and *Ppara*) and mitochondrial biogenesis related genes (*Cox5a*, *Uqcr10*, *Esrra*, *Tfam*, and *Ndufb4*) were also significantly up-regulated in G45a-sh1 cells (Fig. 5b).

We further used the Mito-tracker staining to confirm our results. When examining mitochondrial abundance by using Mito-tracker, we found that Mito-Tracker Red CMXRos was significantly higher in the G45a-sh1 cells (Fig. 5c). Besides, our data from the transmission electron microscope (TEM) indicated morphological and structural changes between G45a-sh1 cells compared to controls (Fig. 5d). Immunofluorescence results showed higher levels of UCP1 expression in *Gadd45a* knockdown cells (Fig. 5e). Collectively, these results suggest that GADD45α deficiency increased the expression of mitochondria related genes and enhanced mitochondrial biogenesis, suggesting GADD45α deletion may have beneficial effects on insulin sensitivity and whole-body energy metabolism.

### GADD45α deficiency inhibits brown adipogenesis and upregulates expression of BAT-selective genes in vivo

To precisely explore the function of GADD45α in BAT, we used the *Gadd45a*^*−/−*^ mice (KO) model to verify our results. Genotyping and real-time polymerase chain reaction (PCR) analyses all confirmed efficient deletion of GADD45α in BAT and WAT (Supplementary Fig. 3a, b), as well as in non-adipose tissues (Supplementary Fig. 3c). The *Gadd45a*^*−/−*^ mice were born at expected Mendelian ratios and were morphologically indistinguishable from their wild-type (WT) littermates (Supplementary Fig. 3d). On the normal chow diet, the *Gadd45a*^*−/−*^ male mice showed similar body weights but higher food intakes compared to WT mice at 8 weeks of age (Supplementary Fig. 3e, f). The BAT masses were similar, while the masses of iWAT from the KO mice were lower than in the WT mice (Supplementary Fig. 3g-i). All other non-adipose tissue masses were not affected by GADD45α deficiency (Supplementary Fig. 3j, k). Our results revealed that GADD45α deficiency did not seem to affect BAT development.

**Fig. 6:**
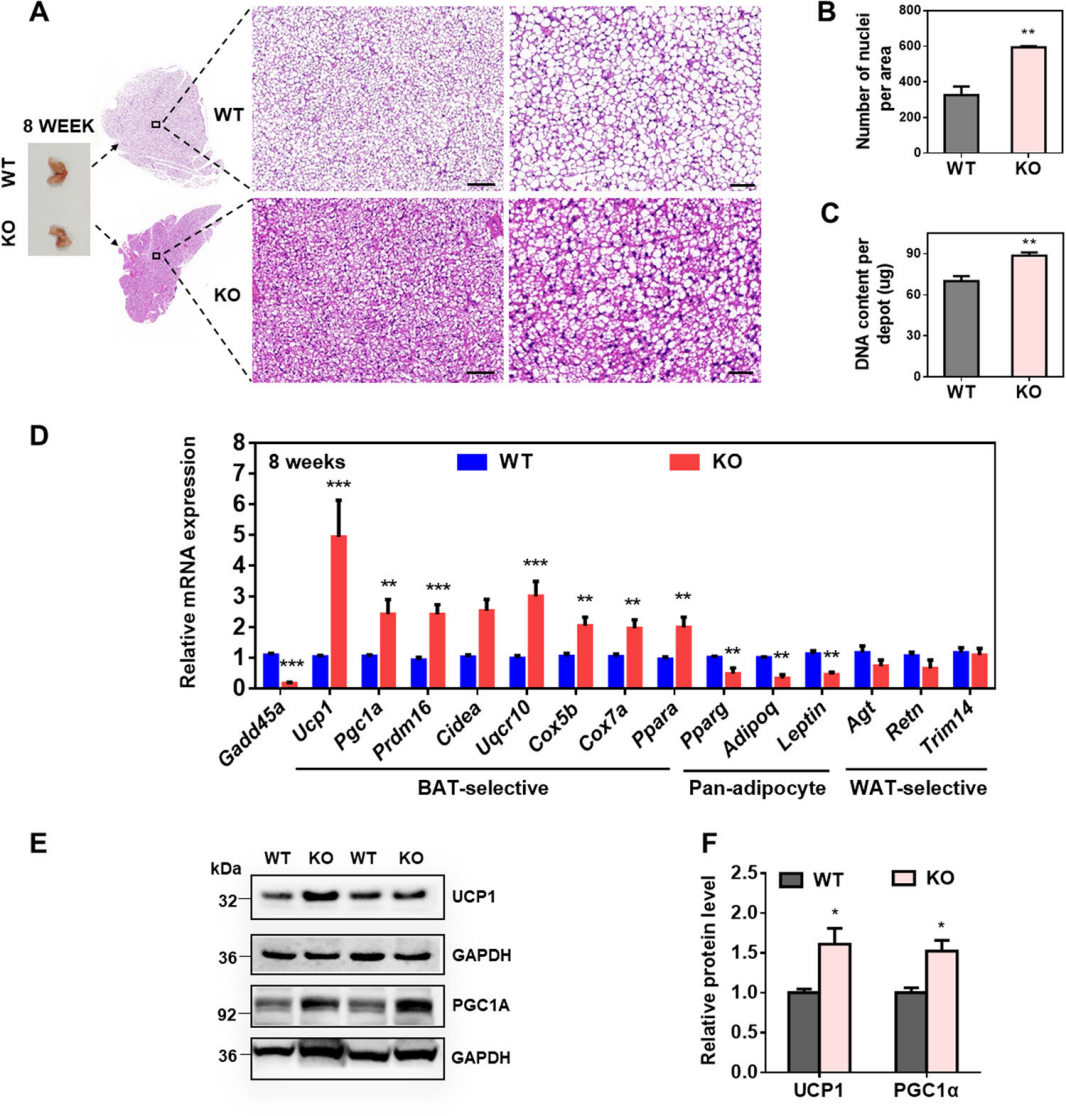
GADD45α deficiency influences BAT adipogenesis and expression of BAT-selective genes in vivo. **a** Representative BAT tissues and H&E staining of BAT sections from WT and KO mice at the age of 8 weeks. Scale bar, 100 and 200 µm, respectively. **b** Number of nuclei per BAT picture in WT and KO mice. Two random images from each mouse were chosen. *n* = 3. **c** Genomic DNA content per BAT depot at the age of 8 weeks. *n* = 4. **d** Relative mRNA expression of pan-adipocyte, BAT- and WAT-selective genes in BAT from WT and KO mice. *n* = 6. **e**, **f** Protein levels of UCP1 and PGC1α in WT and KO mice. Error bars represent SEM, *P < 0.05, **P < 0.01, ***P < 0.001, two-tailed Student’s *t* test.

Interestingly, hematoxylin-eosin (H&E) staining revealed an obvious decrease in adipocyte size in the *Gadd45a*^*−/−*^ BAT compared with WT BAT (Fig. 6a). Nuclear densities (number of nuclei per unit area) were also higher in the KO mice than in the WT mice, confirming smaller adipocyte size in the KO mice (Fig. 6b). In addition, genomic DNA content per BAT depot was higher in the KO mice than in the WT mice (Fig. 6c), suggesting that *Gadd45a*^*−/−*^ BAT contained more cells per depot than the WT BAT deports. Expansion of fat mass can result from increased intracellular lipids and greater adipocyte size (hypertrophy) as well as increased numbers of adipocytes (hyperplasia)^31^. Our *in vivo* and *in vitro* data suggest that GADD45α deficiency may promote proliferation and lipolysis in brown adipocytes.

We further analyzed the expression of adipogenic related genes and found that the mRNA levels of BAT-selective and mitochondrial marker genes, such as *Ucp1*, *Pgc1a*, *Prdm16*, *Uqcr10*, *Cox5b*, *Cox7a*, and *Ppara* were significantly higher in the KO BAT compared with WT BAT (Fig. 6d). By contrast, the mRNA levels of pan-adipocyte genes *Pparg*, *Adipoq* and *Lep* were significantly lower in the KO BAT than WT BAT (Fig. 6d). The expression of the WAT-specific genes *Agt*, *Retn* and *Trim14* was similar between the two genotypes (Fig. 6d). In addition, the KO BAT expressed higher levels of UCP1 and PGC1α protein than the WT BAT (Fig. 6e, f). We also determined the mitochondrial proteins and found that the protein levels of complex CII (SDHB) and CV (ATP5A), key enzymes in oxidative phosphorylation and responsible for energy production^32^, were dramatically elevated in the KO BAT (Supplementary Fig. 4). Overall, our results indicate that GADD45α deficiency affected brown BAT development and upregulated expression of BAT-selective and mitochondrial marker genes.

### GADD45α deficiency enhances insulin sensitivity and energy expenditure

**Fig. 7:**
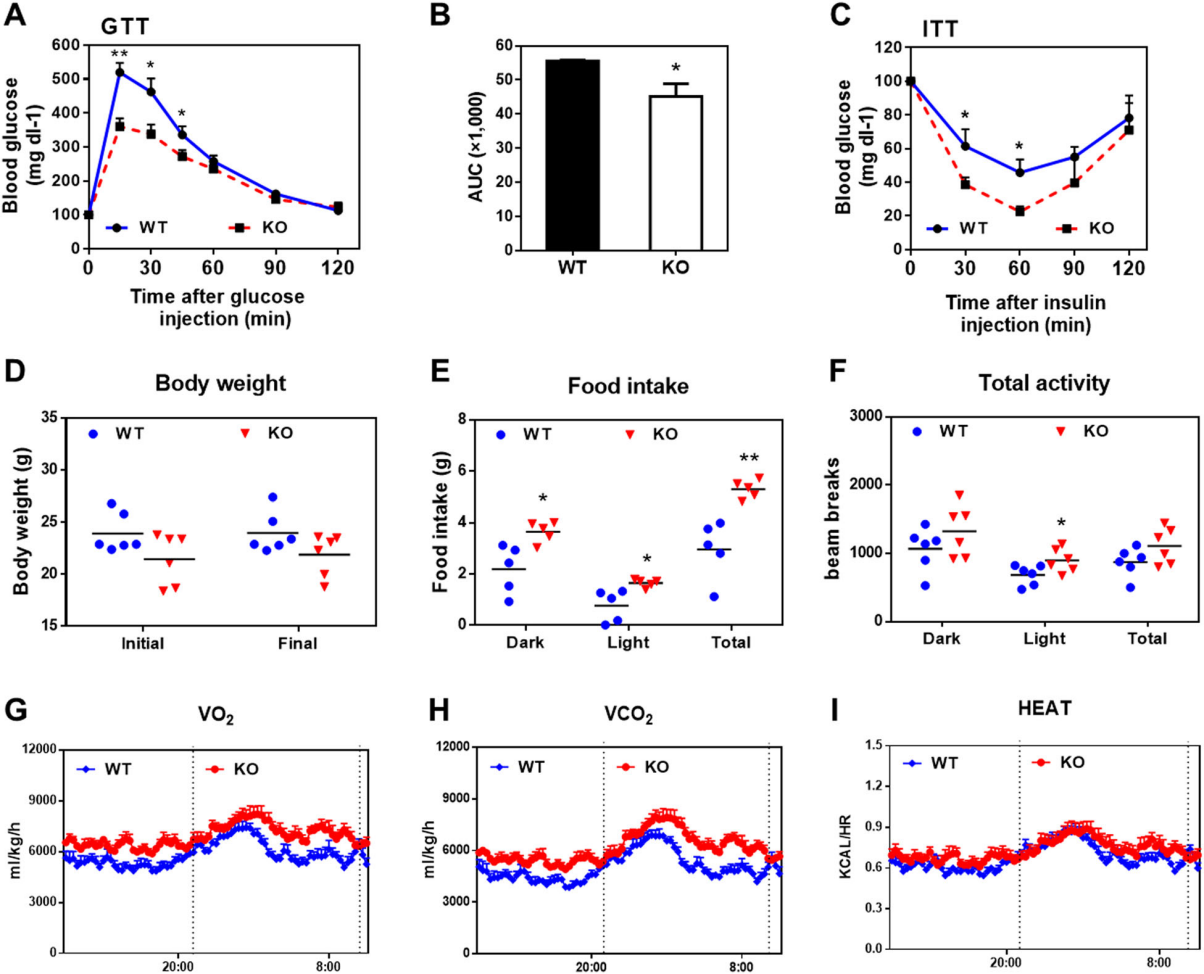
Improved glucose tolerance, insulin sensitivity and higher cellular metabolic rate in *Gadd45a*^*−/−*^ mice. **a**, **b** Blood glucose concentrations and calculated area under curve (AUC) during glucose tolerance tests (GTT) performed in 8-weeks-old WT and KO male mice. *n* = 6. **c** Blood glucose concentrations during insulin tolerance tests (ITT) performed in 8-weeks-old WT and KO male mice. *n* = 6. **d**–**i** Body weight (**d**), food intake (**e**), total activity (**f**), average day and night-time oxygen consumption (VO_2_, **g**), CO_2_ production (VCO_2_, **h**), and heat production (**i**). Error bars represent SEM, *P < 0.05, **P < 0.01, two-tailed Student’s *t* test.

Adipose tissue depots regulate systemic glucose metabolism and insulin sensitivity^33,34^. To determine whether the *Gadd45a*^*−/−*^ mice may have beneficial metabolic health effects, we conducted glucose tolerance tests (GTTs) and insulin tolerance tests (ITTs). Compared to the WT littermates, KO mice had lower blood glucose levels after glucose injection (Fig. 7a, b) and a faster rate of insulin-stimulated glucose clearance (Fig. 7c). To gain further insight into the effect of GADD45α deficiency on whole-body metabolism, metabolic cages were used for the simultaneous measurement of food intake, energy expenditure, heat production and physical activity in the mice. We observed no significant change in body weights between WT and KO mice (Fig. 7d). However, food intake was significantly higher in the KO mice compared to WT mice (Fig. 7e). In addition, the *Gadd45a*^*−/−*^ mice had an increased general activity, higher rates of O_2_ consumption and CO_2_ production (Fig. 7f–h). However, no significant differences in heat production were observed between WT and KO mice (Fig. 7i). Our results demonstrate that GADD45α deficiency improved systemic insulin sensitivity and glucose tolerance, and ameliorated the metabolic profile of mice.

### GADD45α promotes the differentiation of brown adipocytes through interacting with PPARγ and enhancing its transcriptional activity

**Fig. 8:**
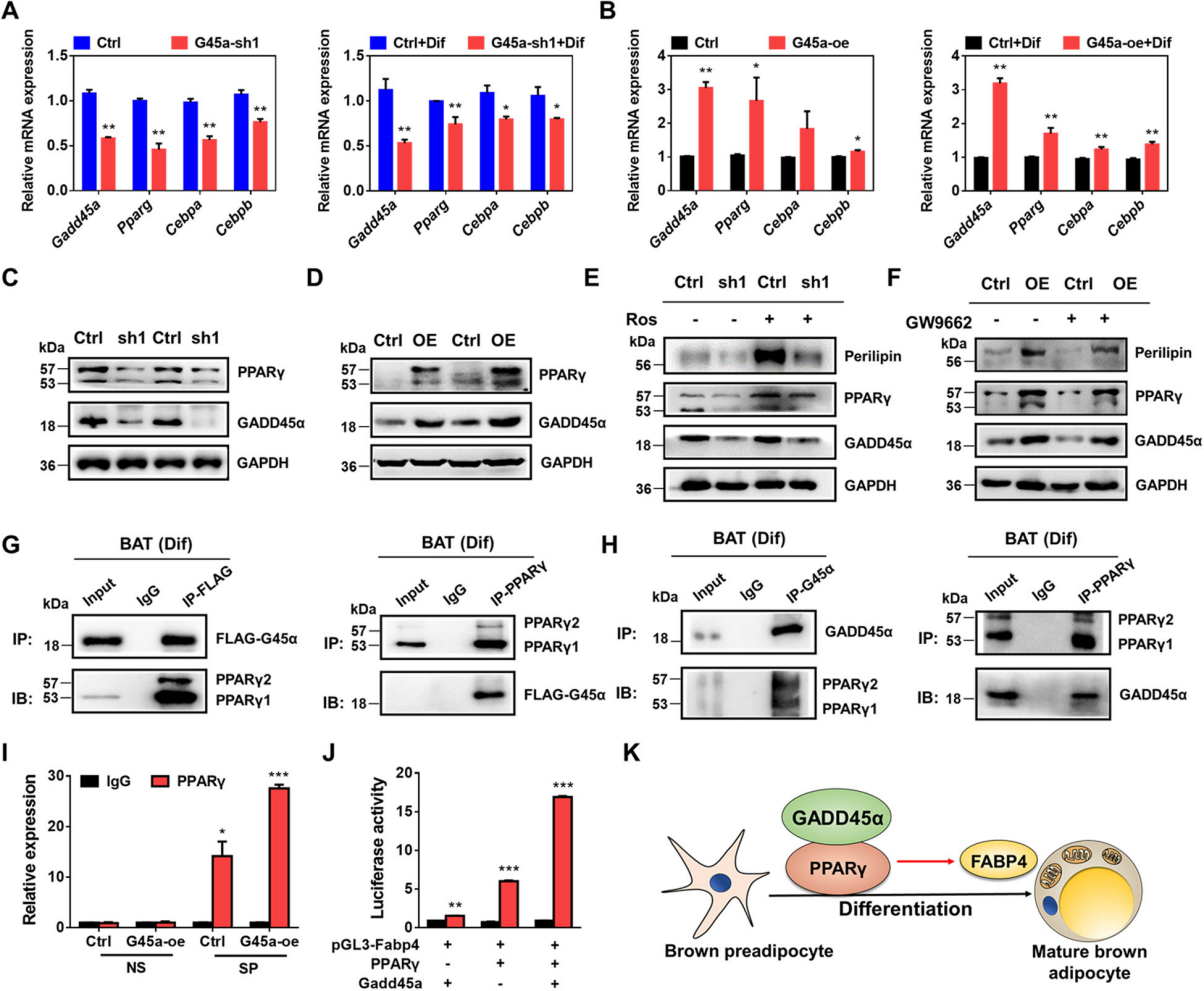
GADD45α binds to PPARγ to enhance its transcriptional activity. **a**, **b** mRNA levels of *Gadd45a*, *Pparg*, and *Cebps* in control and G45a-sh1 cells (**a**), as well as control and G45a-oe cells (**b**). *n* = 6. **c**, **d** Protein levels of PPARγ in G45a-sh1 (**c**) and G45a-oe treated brown adipocytes (**d**) during adipogenic differentiation. **e** Protein levels of PPARγ and Perilipin treated with or without rosiglitazone (Ros). **f** Protein levels of PPARγ and Perilipin treated with or without GW9662. **g** GADD45α interacts with PPARγ. Brown adipocytes were transfected with pCDNA-FLAG-GADD45α and the lysates were immunoprecipitated (IP) with FLAG and PPARγ antibodies and blotted with FLAG and PPARγ antibodies. **h** Endogenously expressed GADD45α interacts with PPARγ. Cell lysates from differentiated brown adipocytes were IP with GADD45α or PPARγ antibodies, and blotted with these antibodies. **i** Brown adipocytes were infected with adenovirus expressing Gadd45a and differentiated. Chromatin immunoprecipitation using a PPARγ specific antibody followed by qPCR amplified by primers flanking FABP4 specific (SP) DNA sequence or nonspecific (NS) sequences in the promoter region of FABP4 gene. *n* = 3. **j** Luciferase assay of 293T cells after co-transfected with the plasmids shown. *n* = 3. **k** Schematic summary illustrating GADD45α regulates brown adipocyte differentiation. Error bars represent SEM, *P < 0.05, **P < 0.01, two-tailed Student’s *t* test.

To closer investigate the cellular and molecular mechanisms through which GADD45α leads to brown adipocyte differentiation, we measured gene expression of the genes involved in regulating adipogenesis based on knockdown or overexpressing of GADD45α (Fig. 8a, b). In brown adipocytes, *Gadd45a* knockdown dramatically decreased the mRNA expression of *Pparg* (Fig. 8a), a key transcription factor that regulates adipogenesis^35,36^, while *Gadd45a* overexpression had the opposite effects (Fig. 8b). Similar results were obtained using western blotting (Fig. 8c, d). GADD45α may thus be associated with PPARγ to regulate BAT adipogenic differentiation. Moreover, rosiglitazone (a PPARγ agonist) treatment rescued the differentiation of the *Gadd45a*-deficient brown adipocytes (Fig. 8e; Supplementary Fig. 5a). By contrast, PPARγ inhibitor GW9662 treatment partly attenuated the lipid droplet formation of *Gadd45a*-oe brown adipocytes (Fig. 8f; Supplementary Fig. 5b, c). From the gene expression and pharmacological rescue data above, we demonstrate that GADD45α positively regulated PPARγ expression at both mRNA and protein level.

We next performed Co-IP experiments to examine whether GADD45α interacts with PPARγ. After GADD45α overexpression in BAT, we found that PPARγ can be pulled down by FLAG-G45α and vice versa (Fig. 8g). Likewise, endogenous interactions between GADD45α and PPARγ were also found in differentiated BAT cells (Fig. 8h). Moreover, immunofluorescence microscopy verified that GADD45α and PPARγ protein had similar subcellular localization in differentiated brown adipocytes (Supplementary Fig. 6a), suggesting GADD45α interacts with PPARγ. To examine whether the interaction regulates the transcriptional activities of PPARγ, we performed a chromatin immunoprecipitation and luciferase reporter assay of FABP4, which is a downstream target of PPARγ and directly regulated by PPARγ^37–39^. The chromatin immunoprecipitation (ChIP) and luciferase reporter assays indicated that PPARγ directly bound to the promoter of FABP4 to enhance FABP4 gene transcription (Supplementary Fig. 6b-d). Notably, co-transfection of GADD45α markedly increased PPARγ transcriptional regulation of FABP4 (Fig. 8i, j), suggesting GADD45α upregulated the transcriptional activity of PPARγ. These results demonstrate that GADD45α promotes brown adipocytes differentiation via interacting with PPARγ to upregulate its transcriptional activity (Fig. 8k).

## Discussion

Our study reveals a novel role of GADD45α in regulating the brown adipogenesis and function. We have provided functional physiological, histological and cellular evidence to demonstrate that GADD45α deficiency improves energy metabolism and mitochondrial biogenesis in mice. We found out that GADD45α deficiency promotes BAT adipocyte proliferation and decreases lipid accumulation. As the GADD45 protein family is considered highly conserved in evolution, we anticipate that our results from mice may be well applicable to humans, although future studies dissecting the role of GADD45α signaling in human adipose tissues will be necessary.

GADD45α is a p53-targeted protein whose expression is induced by several stress agents^12,13^. Previous study demonstrated that stress can lead to metabolic dysfunction and obesity^1^. Using unbiased transcriptomics data analysis, we found that *Gadd45a* mRNA expression was indeed correlated with obesity and may regulate lipid metabolism and brown adipogenesis. Our data indicated that *Gadd45a* knockdown dramatically upregulated the expression of *Ki67* and other cell cycle markers and promoted brown adipocyte proliferation. Vice versa, *Gadd45a* overexpression markedly inhibited the process of brown adipocyte proliferation through inhibiting growth and cell cycle genes. Consistent with our results, GADD45α blocks cell proliferation in hepatocellular carcinoma cells through cell cycle arrest in the G2/M^40,41^. Likewise, *Gadd45a* deletion increased cell proliferation of mouse embryo fibroblasts^22^. In addition, GADD45α acted as an autoimmune suppressor in *vivo* by inhibiting T cell proliferation in response to TCR activation^42^. In our study presented here, GADD45α may be required for adipogenic differentiation in brown pre-adipocytes. *Gadd45a* overexpression promoted brown adipogenesis and lipid accumulation, accompanied by an increased expression of adipogenic genes and decreased glycerol release into the medium. Instead, knockdown of GADD45α inhibited brown pre-adipocytes lipogenesis and facilitated the lipolysis of triglycerides in *vitro*. These observations are suggesting that GADD45α deficiency may regulate the balance between proliferation and differentiation of the precursor cells in BAT.

We found that the *Gadd45a*^*−/−*^ mice had lower iWAT mass and identical BAT masses than the WT mice. The number of brown adipocytes was increased but the cell size was decreased in *Gadd45a*^*−/−*^ mice. This observation suggests that GADD45α deletion may promote BAT proliferation while suppressing differentiation and accumulation of lipids in brown adipocytes in *vivo*. The unchanged BAT mass in *Gadd45a*^*−/−*^ mice may be due to the combinatory effects of i) the increase in cell number and ii) the decrease in cell size. These results are consistent with our above described phenotypes in vitro. Several obesity genes driving food intake and energy expenditure were previously characterized and revealed a homeostatic system for energy metabolism^43,44^. It is interesting that the *Gadd45a*^*−/−*^ mice exhibited improved insulin sensitivity and food intake compared to the WT mice. The beneficial effects on insulin sensitivity and food intake may have been caused due to the upregulated BAT-selective gene expression and the improved mitochondrial biogenesis in the *Gadd45a*^*−/−*^ mice. The increase of food intake (hyperphagia) triggered by fasting is a simple but compelling example of food intake regulation^45^. The higher food intake and physical activity may be due to an alteration in the central nervous system (CNS), critical for normal energy balance^45^. The activation of sympathetic nerves can increase lipolysis and increase thermogenesis in brown adipose tissue, as well as other central and peripheral pathways increasing energy expenditure^46^. Besides, beige adipocyte homeostasis can be bi-directionally converted from and to white adipocytes under the control of environmental cues or innervation^47^.

Previous studies have revealed that sympathetic activation induces heat production by stimulating the lipolysis of cytosolic lipid droplets (LDs) through the β3-adrenergic signaling in BAT. The released fatty acids from glycerol serve as fuel for thermogenesis during cold exposure^48^. Thermogenic respiration is initiated by lipolysis through the cyclic AMP-protein kinase A signaling pathway, and activation of thermogenesis in BAT increases energy expenditure^49,50^. The lipolysis and mobilization of lipid droplets may explain the observed BAT phenotypes in the *Gadd45a*^*−/−*^ mice. It is well known that the thermogenic capacity of BAT depends on UCP1, as well as on the tissue’s high mitochondrial density and oxidative capacity^36,51^. When activated, UCP1 catalyzes the mitochondrial proton gradient, thereby using oxidative respiration to generate heat instead of ATP^35,52^. In addition, both mitochondrial biogenesis and respiration are highly dependent on PGC1α^53^, and ablation of PGC1α leads to reduced mitochondrial content and impaired capacity for cold-induced adaptive, non-shivering thermogenesis^54^. Mice lacking GADD45γ have an impaired UCP1 function and thermogenic response in the cold^55^. GADD45γ overexpression in BAT adipocytes instead enhanced ERRγ-dependent transcription and thermogenesis as well^55^. Consistently, we found that deletion of GADD45α increased both mRNA and protein levels of PGC1α and UCP1 in *vivo* and in *vitro*, suggesting GADD45α deficiency promoted mitochondria biogenesis through upregulating the expression of PGC1α and UCP1. However, the exact mechanism needs being explored in full detail in the near future.

PPARγ is a master regulator of adipocytes differentiation, playing a critical role in systemic lipid and glucose metabolism. We found that GADD45α activates PPARγ expression during brown adipogenesis. GADD45α is a vital mediator in gene-specific activated DNA demethylation during adult stem cell differentiation and white adipogenesis^24,56^. Newly emerging evidence indicates that DNA demethylation plays an important role in regulating PPARγ expression and adipogenesis in intramuscular preadipocytes and 3T3-L1 cells^57,58^. GADD45α could recruit demethylation proteins to CpG island promoters^59^, and the CpG demethylation of the PPARγ promoter may contribute to its expression^58^. Here, we demonstrated that GADD45α interacts with PPARγ and upregulates its transcriptional activity. The activated form of PPARγ is well-accepted to be upstream of FABP4, which is also known as adipocyte protein 2 (aP2) and is involved in the intracellular fatty acid transport and glucose and lipid homeostasis in mature adipocytes^37–39^. The promoter of FABP4 has been widely used in adipocyte-specific recombination in mice^60,61^. In both brown and white adipose tissue, FABP4 marks a distinct population of adipocyte progenitors^62^. Our findings show that PPARγ directly binds to the FABP4 promoter to enhance its transcription. Importantly, we discovered that GADD45α interacts with PPARγ and significantly upregulated the transcriptional regulation of PPARγ on FABP4 expression, thus demonstrating the functional significance of the interaction between GADD45α and PPARγ. This observation is consistent with our result that GADD45α promotes FABP4 expression at both protein and mRNA level in differentiated brown adipocytes.

In summary, our results reveal important regulatory roles of GADD45α in brown adipocytes. We highlight the function of GADD45α in BAT adipogenesis and demonstrate that GADD45α interacts with PPARγ by enhancing its transcriptional activities in brown adipocytes. Our results provide novel insights into the mechanistic role of GADD45α in counteracting obesity and other metabolic diseases.

## Materials and methods

### Animals

All procedures involving mice were approved by the Zhejiang University Animal Care and Use Committee. *Gadd45a*^*−/−*^ mice^22^ were directly contributed from Professor Albert J. Fornace Jr. (Gene Response Section, DBS, National Cancer Institute, USA) and were maintained on a C57BL/6 background. All mice used in this study, the *Gadd45a*^*−/−*^ mice and their WT littermate controls, were produced from intercrossing of *Gadd45a*^*+/−*^ mice obtained from Hangzhou Normal University. Male mice were single housed in the animal facility with free access to water and standard rodent chow food. The age of the mice was between 8 and 10 weeks in the experiments. PCR genotyping was carried out as described by the supplier. Food intakes were measured by weighing total individual food consumption once per week.

### Blood glucose measurements

For GTT, mice were given an i.p. injection of 100 mg ml^−1^ d-glucose (2 g kg^−1^ body weight) after overnight fasting^3^, and tail blood glucose concentrations were measured by a glucometer (Accu-Check Active, Roche). For ITT, mice were fasted for 4 h before the i.p. administration of human insulin (Santa Cruz) (0.75 U per kg body weight)^3^, and tail blood glucose concentrations were monitored. For both GTT and ITT, each mouse was singly caged with blinded cage number and random orders.

### Indirect calorimetry study

Oxygen consumption (VO_2_), carbon dioxide production (VCO_2_), respiratory exchange ratios and heat production were measured using an indirect calorimetry system (Oxymax, Columbus Instruments), installed under a constant environmental temperature (22 or 30 °C) and a 12-h light (06:00–18:00 h), 12-h dark cycle (18:00–06:00 h). All mice had free unlimited access to food and drinking water. The raw data were normalized to the lean mass of the mice.

### Cell transfection, plasmids, and RNA knockdown

The expression of *Gadd45a* was inhibited by small hairpin RNA (shRNA) interference. sh-*Gadd45a* and its corresponding negative control were purchased from Vigene Biosciences (Shandong, China). sh-*Gadd45a* lentiviral particles were produced by transfecting 293T cells with three plasmids-pMD2.G, psPAX2, and Lenti-sh-*Gadd45a* or Lenti-sh-Luciferase (sh-Control) vectors. The sequences for shRNA were as follows: shRNA1 5′-AACGTCGACCCCGATAACGTG-3′, shRNA2 5′-CCCGTGATTAATCTCCCGG-3′, shRNA3 5′-GCTCGGAGTCAGCGCACCA-3′. For *Gadd45a* knockdown, cells were infected with Lenti-sh-*Gadd45a* virus. The knockdown of *Gadd45a* was confirmed by quantitative qRT-PCR and western blotting after 48 h post virus infection. The BAT cell line (20–30%) was infected by *Gadd45a* or scramble lentivirus, and then the stable expressing shRNA cells were selected by puromycin (2.5 μg/ml). For overexpression, control adenovirus and *Gadd45a* overexpression adenovirus were purchased from Vigene Biosciences (Shandong, China).

### Cell culture and adipogenic differentiation

Primary BAT stromal vascular fraction (SVF) cells were isolated using collagenase digestion followed by density separation. Briefly, the interscapular brown adipose (BAT) was minced and digested in 1.5 mg/ml collagenase at 37 °C for 0.5 and 1 h, respectively. The digestions were terminated with Dulbecco’s modification of Eagle’s medium (DMEM) containing 10% fetal bovine serum (FBS) (Gibco, CA, USA), and filtered through 100 µm filters to remove connective tissues and undigested trunks of tissues. Cells were then centrifuged at 450 g for 5 min to separate the SVF cells. The freshly isolated SVF cells were seeded and cultured in growth medium containing DMEM, 20% FBS, 1% penicillin/streptomycin (Invitrogen) at 37 °C with 5% CO_2_ for 3 days, followed by feeding with fresh medium every 2 days. The BAT cell lines, were cultured under the same conditions as SVF cells. For BAT SVF cells adipogenic differentiation, the cells were induced with induction medium (IM) contains DMEM, 10% FBS, 2.85 mM insulin, 0.3 mM dexamethasone (DEXA) and 0.63 mM 3-isobutyl-methylxanthine (IBMX) for 4 days on confluence and then differentiated in differentiation medium (DM) contains DMEM, 10% FBS, 200 nM insulin and 10 nM T3 for 2 days until adipocytes mature. To avoid a cell density effect on adipogenic differentiation, cells were induced to differentiate when they reach 90% confluence.

### Oil red O staining

Cultured cells were washed with PBS and fixed with 4% formaldehyde for 15 min at room temperature. Then the cells were stained using the Oil red O working solutions containing 6 ml Oil red O stock solution (5 g l^−1^ in isopropanol) and 4 ml ddH_2_O for 30 min. After staining, the cells were washed with 60% isopropanol in PBS and pictured. Oil red O dye were extracted from stained adipocytes with 100% isopropanol, and the Oil red signal were quantified by measuring the optical density at 490 nm (OD 490).

### Glycerol release measurements

Glycerol release was assessed using the free glycerol reagens (Sigma-Aldrich, USA). For in vitro lipolysis, glycerol release from differentiated adipocytes was measured as previously described^63^. The results are expressed in µg glycerol released per mg protein.

### H&E and immunostaining

Adipose tissues were fixed in 4% formalin for 24 h at room temperature. Then the tissues were embedded into paraffin and cut at 4-µm thick slices. For H&E staining, the sections were deparaffinized, rehydrated and the nuclei stained with haematoxylin for 15 min. Sections were then rinsed in running tap water and stained with eosin for 1 min, dehydrated and mounted. Whole-slide digital images were collected at ×20 magnification with an Aperio Scan Scope slide scanner (Aperio, Vista, CA). Scanned images of H&E staining were analyzed by Photoshop CS3 to calculate numbers of nuclei. For immunostaining, the sections were blocked with blocking buffer containing 5% goat serum, 2% BSA, 0.2% triton X-100 and 0.1% sodium azide in PBS for 1 h after deparaffinized and antigen retrieval. Then the samples were incubated with Ki67 (Abcam, ab16667, 1:500), PPARγ (C26H12, 1:500) and GADD45α (sc-6850, 1:200) primary antibodies diluted in blocking buffer overnight. After washing with PBS, the samples were incubated with secondary antibodies and DAPI for 45 min at room temperature. Fluorescent images were captured as single-channel grayscale images using a Leica DM 6000B fluorescent microscope with a ×20 objective (NA 0.70).

### Mito-tracker and bodipy staining

Control and *Gadd45a* knockdown (G45a-sh1) or *Gadd45a* overexpressing (G45a-oe) cells were incubated for 15 minutes with 20 nM MitoTracker^®^ Red CMXRos (Invitrogen). Then cells were washed with PBS for 3 times, then were added fresh DMEM medium and pictures were taken. Intracellular lipids were visualized by staining with 0.5 nM BODIPY FL (Invitrogen) for 10 min. Cells were fixed afterwards with 4% paraformaldehyde and were observed by fluorescence microscopy.

### Transmission electron microscopy (TEM)

TEM assay was performed as described^64^. Electron photomicrographs were taken from cell ultrastructures under a transmission electron microscopy (Hitachi, H-7650).

### Cell growth rate

Cell growth rates were determined as described previously^65^. BAT cells were seeded in six-well plates (1 × 10^4^ cells per well) and cultured under standard adipocyte conditions with or without drug treatment. The cells were harvested and counted using a hemocytometer.

### Total RNA extraction and real-time PCR

Total RNA was extracted from cells or tissues using Trizol Reagent (Invitrogen, CA, USA) and following the manufacturer’s instructions. The purity and concentration of total RNA were measured by a spectrophotometer (Nanodrop 2000; Thermo Fisher Scientific) at 260 and 280 nm. Absorption rates (260/280 nm) of all samples were between 1.8 and 2.0. Then the first-strand cDNA was synthesized using random primers with a reverse transcription kit (Invitrogen, USA). Real-time PCR was carried out with a Roche Lightcycler 480 PCR System using SYBR Green Master Mix and gene-specific primers (Table S1). The 2^−ΔΔCT^ method was used to analyze the relative changes in gene expression normalized against 18 S rRNA as internal control.

### RNA-seq analysis

RNA extraction and RNA-seq analysis were performed by Novogene Bioinformatics Institute (Beijing, China). Sequencing libraries were generated from 1 µg total RNA using NEBNext^®^ UltraTM RNA Library Prep Kit for Illumina^®^ (NEB, USA), following manufacturer’s recommendations. The libraries were then quantified and pooled. Paired-end sequencing of the library was performed on the HiSeq XTen sequencers (Illumina, San Diego, CA). FastQC (version 0.11.2) was used for evaluating the quality of sequenced data. Gene expression values of the transcripts were computed by StringTie (version 1.3.3b). The TPM eliminated the influence of gene lengths and sequencing discrepancies to enable direct comparison of gene expression between samples. Differential expression analysis of two groups was performed using the DESeq2 R package (1.16.1). Genes were considered as significantly differentially expressed if *p* value < 0.001 and |foldchange | > 1.5. GO enrichment analysis of differentially expressed genes was implemented by the clusterProfiler R package.

### Co-IP assay

Total protein was extracted from differentiated brown adipocytes. The lysate was precleared with protein A/G agarose at 4 °C for 1 h. Then 2 mg of primary antibody anti-GADD45α (sc-6850, Santa), anti- PPARγ (C26H12, CST) anti-FLAG (M20008S, Abmart) was added into lysate contains 500 mg total protein and rotating at 4 °C overnight. In the next morning the protein A/G agarose was added and rotated for 2 h. The samples were washed with cold PBS for three times and collected for western blotting.

### Protein extraction and western blotting analysis

Total protein was isolated from cells or tissues using RIPA buffer. Protein separation and western blot analysis were conducted as described earlier^66^. GADD45α antibody (GTX54090, 1:1000) was from GeneTex. UCP1 (ab10983, 1:2000) and Perilipin (ab61682, 1:2,000) were from Abcam. FABP4 (E71703-98, 1:2000), GAPDH (EM1101, 1:5000) was from HuaBio. PPARγ (C26H12, 1:1000) was from Cell Signaling Technology (CST). Cocktail (45-8099, 1:2000) is from Thermo Fisher Scientific. PGC1α (sc-13067) was from Santa Cruz Biotechnology (Santa Cruz). The horseradish peroxidase (HRP)-conjugated secondary antibody (anti-rabbit IgG, 111-035-003 or anti-mouse IgG; 115-035-003, Jackson ImmunoResearch) was diluted 1:10,000. Immunodetection was performed using enhanced chemiluminescence western blotting substrate (Google Biotechnology, Wuhan, Hubei, China) and detected by ChemiScope3500 Mini System.

### ChIP assay

Brown preadipocytes were seeded on to 10 cm plates and grown to confluence. Cells were harvested 6 days after adipogenic differentiation. Protein–DNA complexes were cross-linked using 1% formaldehyde for 10 min and the cross-linking was then quenched with the addition of 125 mM glycine for 5 min. Samples were washed twice with cold PBS and placed in SDS lysis buffer containing 20 mM Tris, 0.1% SDS, 1% Triton-100, 150 mM NaCl, 1 Mm EDTA and protease inhibitor. The samples were further sonicated and diluted for IP with the indicated antibodies PPARγ (C26H12, 1:100) or rabbit IgG (sc-2027, Santa Cruz) and incubation at 4 °C overnight. Then, the immunoprecipitates were eluted and reverse crosslinked overnight at 65 °C. DNA was purified using the Cycle Pure kit (Omega Bio-Tek), and qPCR was performed.

### Luciferase assay

HEK293T cells were seeded into 24-well plates for 24 h and then transfected with different plasmids using Lipofectamine 2000 (Invitrogen, USA). The pGL3-FABP4 promoter luciferase plasmid was generated. For transfection of each well, 80 ng Renilla plasmid (pRL-TK), 250 ng pGL3-FABP4 and 300 ng pcDNA-GADD45A plasmid (or its blank control plasmid) and/or 300 ng pcDNA-FLAG-PPARγ (or its blank control plasmid) were co-transfected following the manufacturer’s instructions. Cells were harvested 36 h after transfection and analyzed with the Dual-Luciferase Reporter Assay System (Promega).

### Statistical analysis

Data were presented as means ± SEM from at least three independent experiments. GraphPad (Prism 6) was used for data analyses. Comparisons were made by two-tailed Student’s *t* tests. Differences were considered significant at *P* < 0.05.

## Supplementary information

Supplementary table 1

Supplementary figure legends

Supplementary figure 1

Supplementary figure 2

Supplementary figure 3

Supplementary figure 4

Supplementary figure 5

Supplementary figure 6
