# Supplementary material for: GADD45α drives brown adipose tissue formation through upregulating PPARγ in mice
Source: Cell Death Dis. 2020 Jul 27;11(7):585. doi: 10.1038/s41419-020-02802-5 (PMC7385159; doi:10.1038/s41419-020-02802-5)
Supplement: Supplementary file 1 — Supplementary table 1 [file 41419_2020_2802_MOESM1_ESM.docx]

**Supplementary Table 1. The primer sequence of real-time PCR**

| Primer name | Sequence (5' -> 3') |
| --- | --- |
| 18s-F | TTCTGGCCAACGGTCTAGACAAC |
| 18s-R | CCAGTGGTCTTGGTGTGCTGA |
| Adipoq-F | TGTTCCTCTTAATCCTGCCCA |
| Adipoq-R | CCAACCTGCACAAGTTCCCTT |
| Agt-F | TCTCCTTTACCACAACAAGAGCA |
| Agt-R | CTTCTCATTCACAGGGGAGGT |
| Fabp4-F | ACCGCAGACGACAGGAA |
| Fabp4-R | CTCATGCCCTTTCATAAAC |
| Ccnd1-F | CGTGCAGAAGGAGATTGTGC |
| Ccnd1-R | CAGGAAGCGGTCCAGGTAGT |
| Ccnd3-F | CCATCCATGATCGCCACAGG |
| Ccnd3-R | GGGCAGCTTCGATCTGTTCC |
| Cdkn1a-F | CCTTGTCGCTGTCTTGCACT |
| Cdkn1a-R | CAATCTGCGCTTGGAGTGAT |
| Cdkn1c-F | GCTGAAGGACCAGCCTCTCT |
| Cdkn1c-R | GTTCGACGCCTTGTTCTCCT |
| Cdk5r1-F | CGGTGTCAGCGAAGAAGAAG |
| Cdk5r1-R | GACAGGTTGGCACAGGACAG |
| Cebpa-F | CAAGAACAGCAACGAGTACCG |
| Cebpa-R | GTCACTGGTCAACTCCAGCAC |
| Cebpb-F | ACCGGGTTTCGGGACTTGA |
| Cebpb-R | CCCGCAGGAACATCTTTAAGTGA |
| Cidea-F | TGACATTCATGGGATTGCAGAC |
| Cidea-R | GGCCAGTTGTGATGACTAAGAC |
| Cox5b-F | TTCAAGGTTACTTCGCGGAGT |
| Cox5b-F | CGGGACTAGATTAGGGTCTTCC |
| Cox7a-F | GCTCTGGTCCGGTCTTTTAGC |
| Cox7a-F | GTACTGGGAGGTCATTGTCGG |
| Esrra-F | CTCAGCTCTCTACCCAAACGC |
| Esrra-R | CCGCTTGGTGATCTCACACTC |
| Fabp4-F | AAGGTGAAGAGCATCATAACCCT |
| Fabp4-R | TCACGCCTTTCATAACACATTCC |
| Fasn-F | CGGTCTGGAAAGCTGAAGGATC |
| Fasn-R | GGAGTGAGGCTGGGTTGATACC |
| Gadd45a-F | ACGTGGTACTGTGCCTGCTG |
| Gadd45a-R | CGCAGGATGTTGATGTCGTT |
| Gadd45b-F | AGAAGATGCAGGCGGTGACT |
| Gadd45b-R | AGGCACAAGACCACGCTGT |
| Gadd45g-F | ACGTTGATTCAGGCGTTCTG |
| Gadd45g-R | GGTCCTTCCATGTGTCCTCA |
| Ki67-F | CTGGTCACCATCAAGCGGAG |
| Ki67-R | CAATACTCCTTCCAAACAGGCAG |
| Leptin F | GAGACCCCTGTGTCGGTTC |
| Leptin R | CTGCGTGTGTGAAATGTCATTG |
| Mterf1-F | AGAGGCGGAAGTGAAAGGTG |
| Mterf1-R | AAGTTGCTCAGCAGGTCCTC |
| Nduf4-F | CGGCTTAAACGGGAGTATCTGC |
| Nduf4-R | CCTGCCACAGCTCCTAAAAGTGAG |
| Pgc1a-F | TATGGAGTGACATAGAGTGTGCT |
| Pgc1a-R | CCACTTCAATCCACCCAGAAAG |
| Ppara-F | AGAGCCCCATCTGTCCTCTC |
| Ppara-R | ACTGGTAGTCTGCAAAACCAAA |
| Pparg-F | TCGCTGATGCACTGCCTATG |
| Pparg-R | GAGAGGTCCACAGAGCTGATT |
| Prdm16-F | CCACCAGCGAGGACTTCAC |
| Prdm16-R | GGAGGACTCTCGTAGCTCGAA |
| Retn-F | AAGAACCTTTCATTTCCCCTCCT |
| Retn-R | GTCCAGCAATTTAAGCCAATGTT |
| Tfam-F | CCAGGAGGCAAAGGATGATTCG |
| Tfam-R | CCAACTTCAGCCATCTGCTCTTCCC |
| Trim14-F | GTGCGTGTGCAGAAGCTAATC |
| Trim14-R | CTGCGTAAACCTTGAGCCTTT |
| Ucp1-F | AGGCTTCCAGTACCATTAGGT |
| Ucp1-R | CTGAGTGAGGCAAAGCTGATTT |
| Ucp2-F | CAGGTCACTGTGCCCTTACCAT |
| Ucp2-R | CACTACGTTCCAGGATCCCAAG |
| Ucp3-F | GCCTTCTCTCTCGGAGGTTT |
| Ucp3-R | GCAGATGGAAGACTGAAGGC |
| Uqcr10-F | CCACCTTTGCCCTCACCATC |
| Uqcr10-R | CTCATACTTGTGCTTTATATGTTTCCACAG |
