# Supplementary material for: GADD45α drives brown adipose tissue formation through upregulating PPARγ in mice
Source: Cell Death Dis. 2020 Jul 27;11(7):585. doi: 10.1038/s41419-020-02802-5 (PMC7385159; doi:10.1038/s41419-020-02802-5)
Supplement: Supplementary file 2 — Supplementary figure legends [file 41419_2020_2802_MOESM2_ESM.docx]

**Supplementary Figure Legends**

**Supplementary Fig. 1 Overexpression of *Gadd45a* inhibits proliferation of brown adipocytes.** (**a**, **b**) Brown adipocytes stained with proliferating marker Ki67 (red) and DAPI (blue) in control and G45a-oe cells (**a**), and the percentage of Ki67^+^ cells (**b**). (**c**) Colony assay comparing growth of control and G45a-oe cells. (**d**) Growth curve analysis of the cultured control and G45a-oe cells. Error bars represent SEM, * *P*<0.05, ** *P*<0.01, ****P*<0.001, two-tailed Student’s t-test. Scale bars: 100 µm.

**Supplementary Fig. 2 *Gadd45a* knockdown promotes lipolysis in brown adipocytes.** (**a**, **b**) Glycerol release was determined in the culture media as described in Materials and Methods after *Gadd45a* knockdown (**a**) or while overexpressing (**b**). The results are expressed as µg of released glycerol per µg of protein and are the mean ± SEM. of three independent experiments. * *P*< 0.05, ** *P*< 0.01, two-tailed Student’s t test.

**Supplementary Fig. 3 The phenotype of *Gadd45a^-/-^*** mouse **model.** (**a**) Genotyping of wild-type (WT), [heterozygote](file:///C:/Users/YWJ/AppData/Local/youdao/dict/Application/8.5.1.0/resultui/html/index.html#/javascript:;) (HE) and homozygote mice (KO). (**b**, **c**) Efficient reduction of Gadd45a mRNA levels in adipose (**b**) and non-adipose tissues (**c**) of the *Gadd45a^-/-^* mice. n=5. (**d**) Representative images of WT and KO mice. (**e**, **f**) Body mass (**e**, Female, n=7, Male, n=12) and food intake (**f**, n=6) of WT and KO mice. (**g**) Representative images of BAT and WAT depots in WT and KO mice. (**h-k**) BAT mass (**h**), WAT mass (**i**), muscle mass (**j**) and other organs weight (**k**) in WT and KO mice (2-3 months old). n=8. Error bars represent SEM, **P*<0.05, ***P*<0.01, ****P*<0.001, two-tailed Student’s t-test. TA, tibialis anterior muscle; Sol, soleus muscle; EDL, extensor digitorum longus muscle; Gas, gastrocnemius muscle; Hrt, heart; Kid, kidney; Liv, liver; Spl, spleen; Lun, lung.

**Supplementary Fig. 4 GADD45α regulates brown adipocyte mitochondrial biogenesis.** (**a**, **b**) The protein levels of ETC (electron transport chain) complexes in WT and KO BAT sections. Error bars represent SEM, **P*<0.05, ***P*<0.01, ****P*<0.001, two-tailed Student’s t-test.

**Supplementary Fig. 5 GADD45α affects differentiation of brown adipocytes through** **PPARγ.** (**a**) Oil Red O staining of G45a-sh1 BAT cells treated with or without rosiglitazone (Ros). (**b**, **c**) Oil Red O staining (**b**) and mRNA levels of adipogenic genes (**c**) in G45a-oe BAT cells treated with or without GW9662. n=6. Error bars represent SEM, **P*< 0.05, ***P*< 0.01, ****P*<0.001, two-tailed Student’s t-test. Scale bars: 200 µm.

**Supplementary Fig. 6** **GADD45α interacts with PPARγ and enhances its transcriptional regulation on Fabp4 expression.** (**a**) GADD45α (Green) overexpressing brown adipocytes stained with PPARγ (Red) antibody to label the localization. Scale bars: 50 µm. (**b, c**) Chromatin immunoprecipitation assay results. Brown adipocytes were differentiated. Chromatin immunoprecipitation assay was performed as previous described. n=3. (**d**) Luciferase assay of 293T cells after co-transfection of pGL3-Fabp4 plasmid and pcDNA-FLAG-PPARγ (or control vectors). n=3. Error bars represent SEM, * *P*< 0.05, ** *P*< 0.01, *** *P*< 0.001, two-tailed Student’s t test.
