# Supplementary figures and images for: GADD45α drives brown adipose tissue formation through upregulating PPARγ in mice
Source: Cell Death Dis. 2020 Jul 27;11(7):585. doi: 10.1038/s41419-020-02802-5 (PMC7385159; doi:10.1038/s41419-020-02802-5)

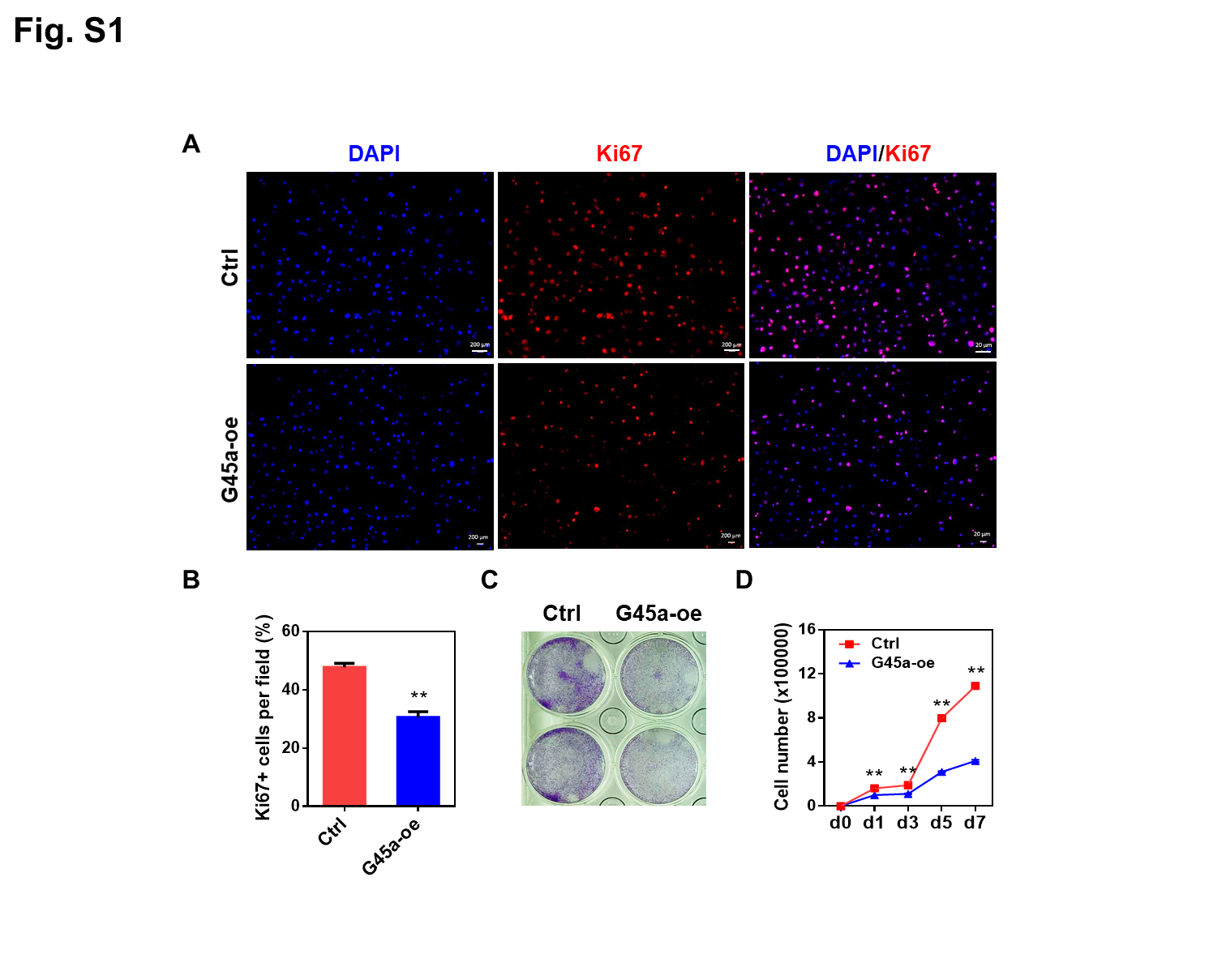

Supplement: Supplementary file 3 — Supplementary figure 1 [file 41419_2020_2802_MOESM3_ESM.tif]

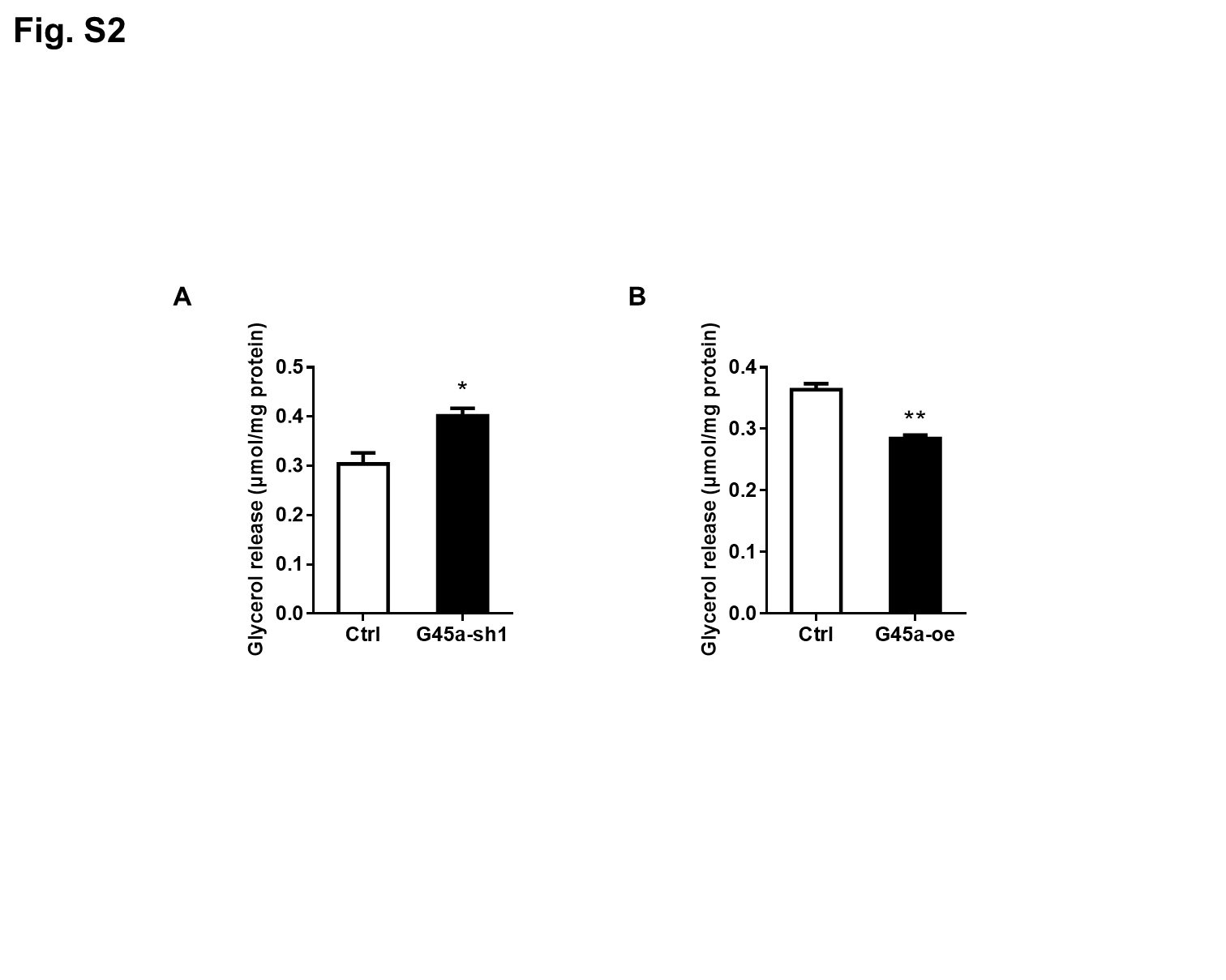

Supplement: Supplementary file 4 — Supplementary figure 2 [file 41419_2020_2802_MOESM4_ESM.tif]

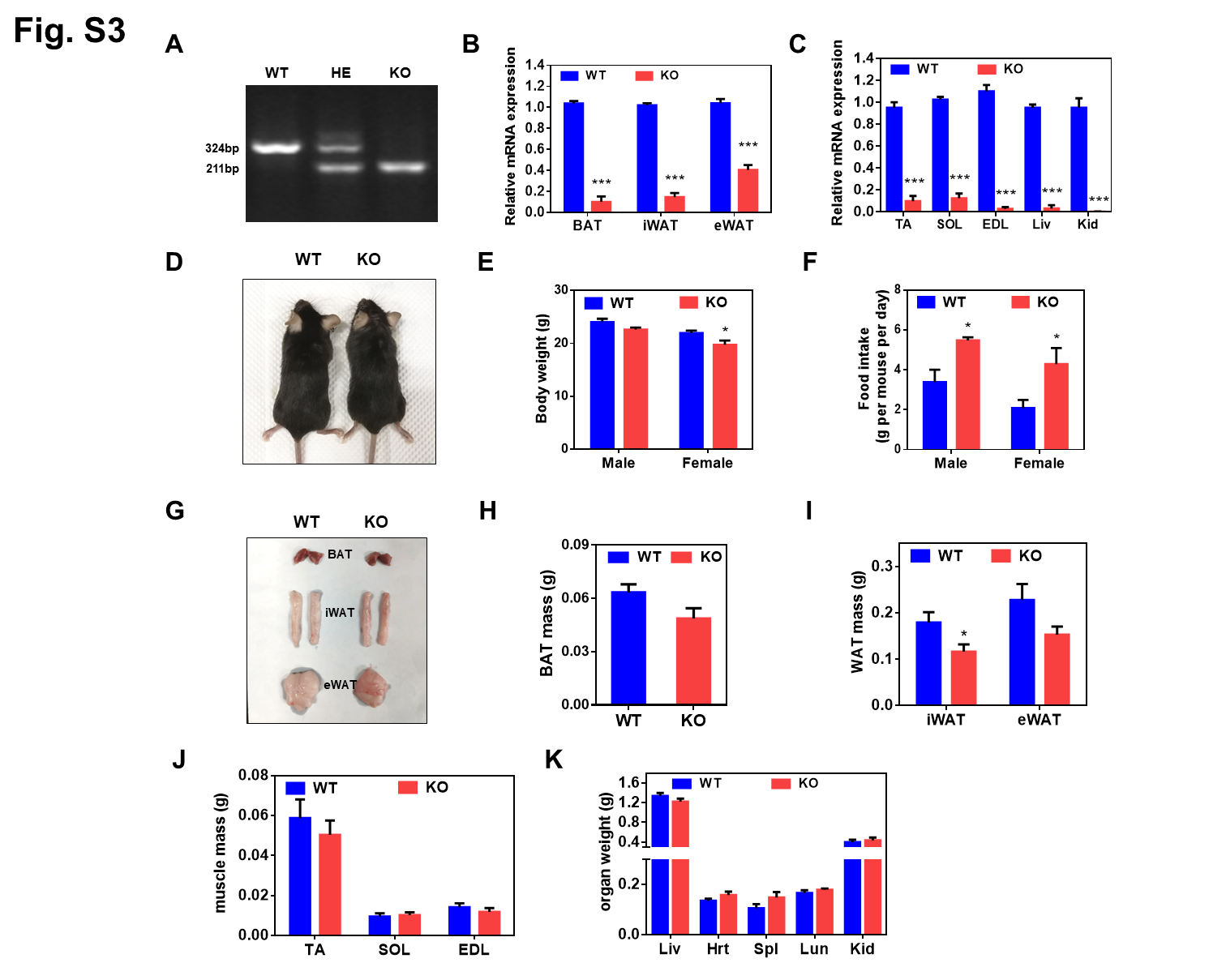

Supplement: Supplementary file 5 — Supplementary figure 3 [file 41419_2020_2802_MOESM5_ESM.tif]

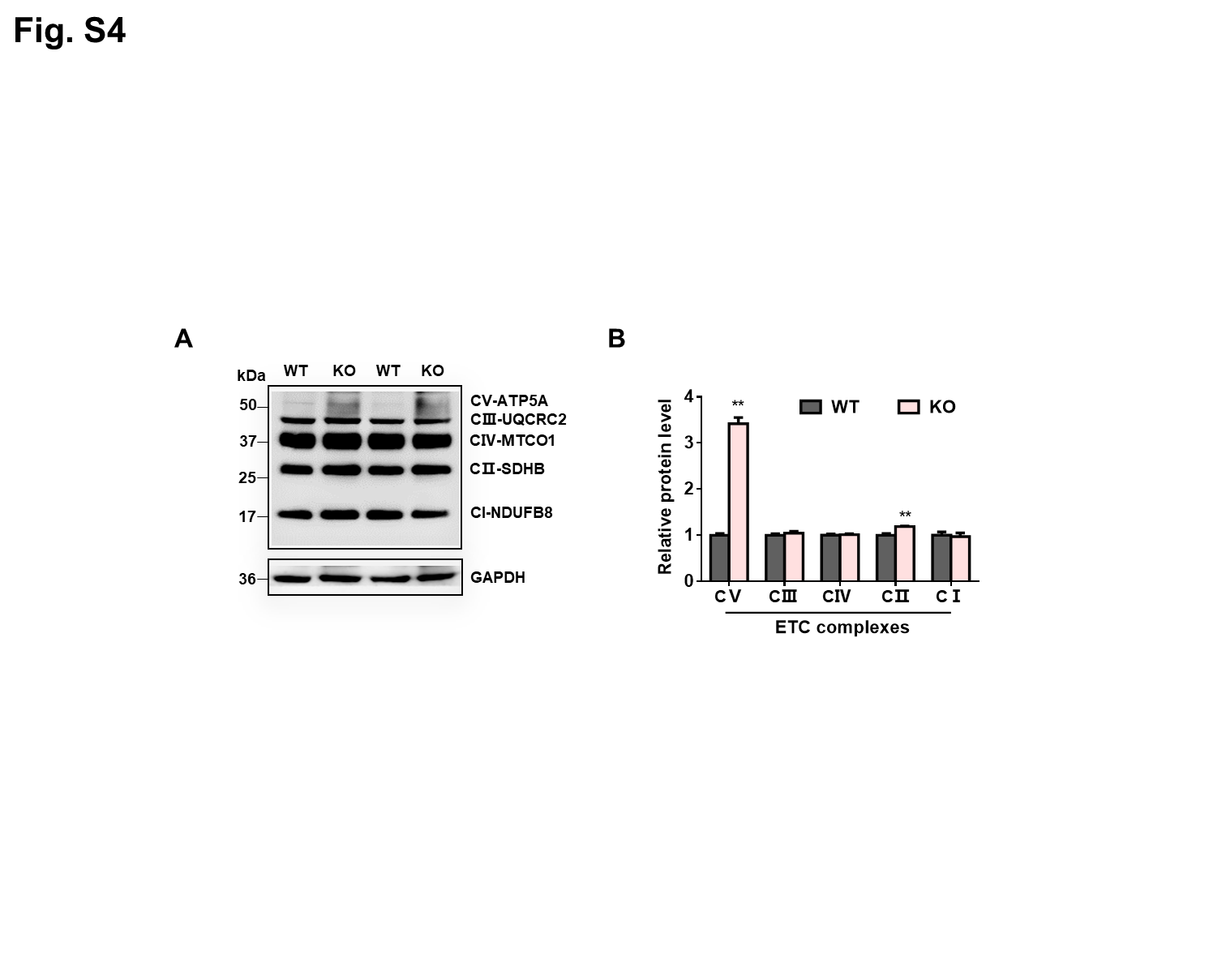

Supplement: Supplementary file 6 — Supplementary figure 4 [file 41419_2020_2802_MOESM6_ESM.tif]

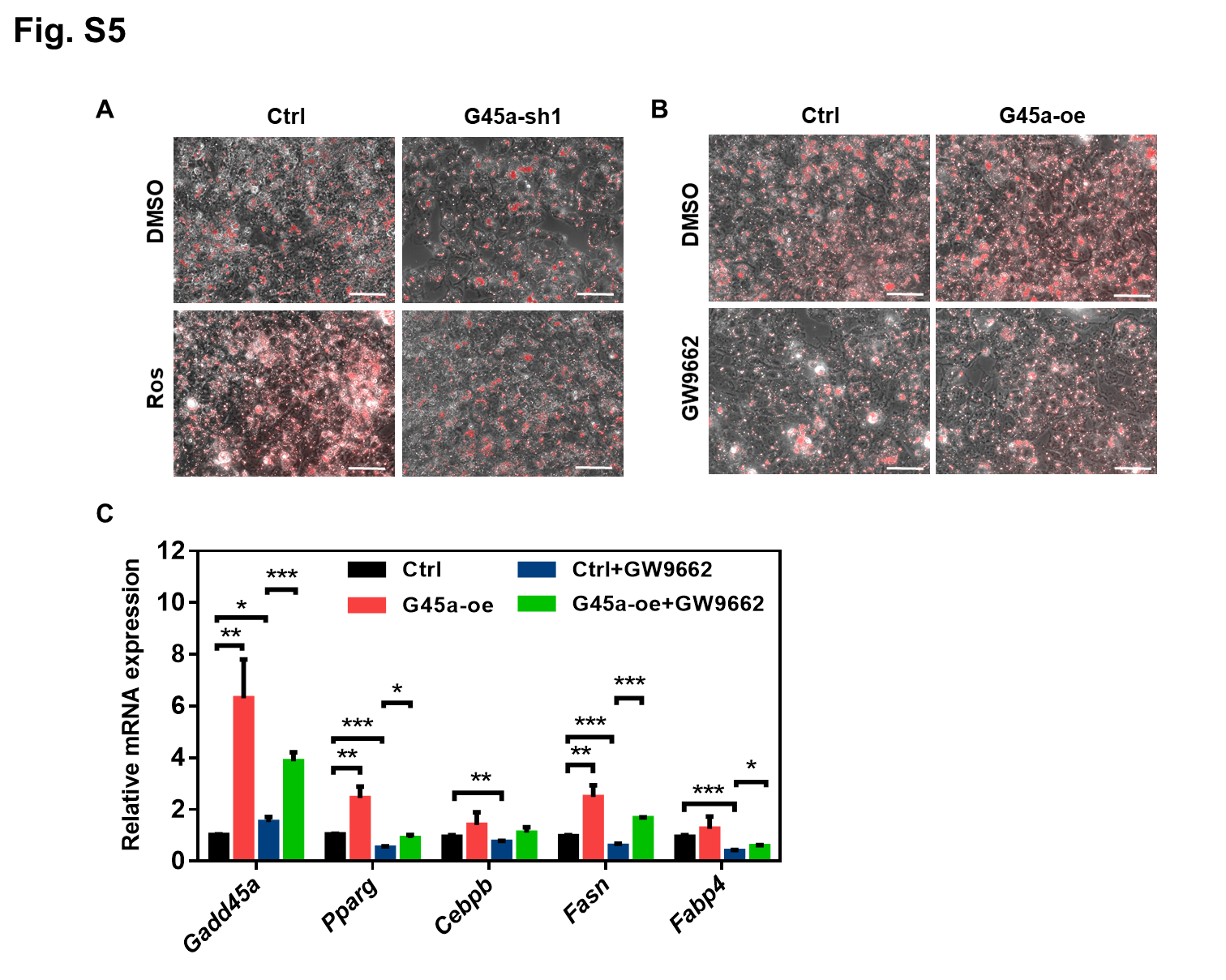

Supplement: Supplementary file 7 — Supplementary figure 5 [file 41419_2020_2802_MOESM7_ESM.tif]

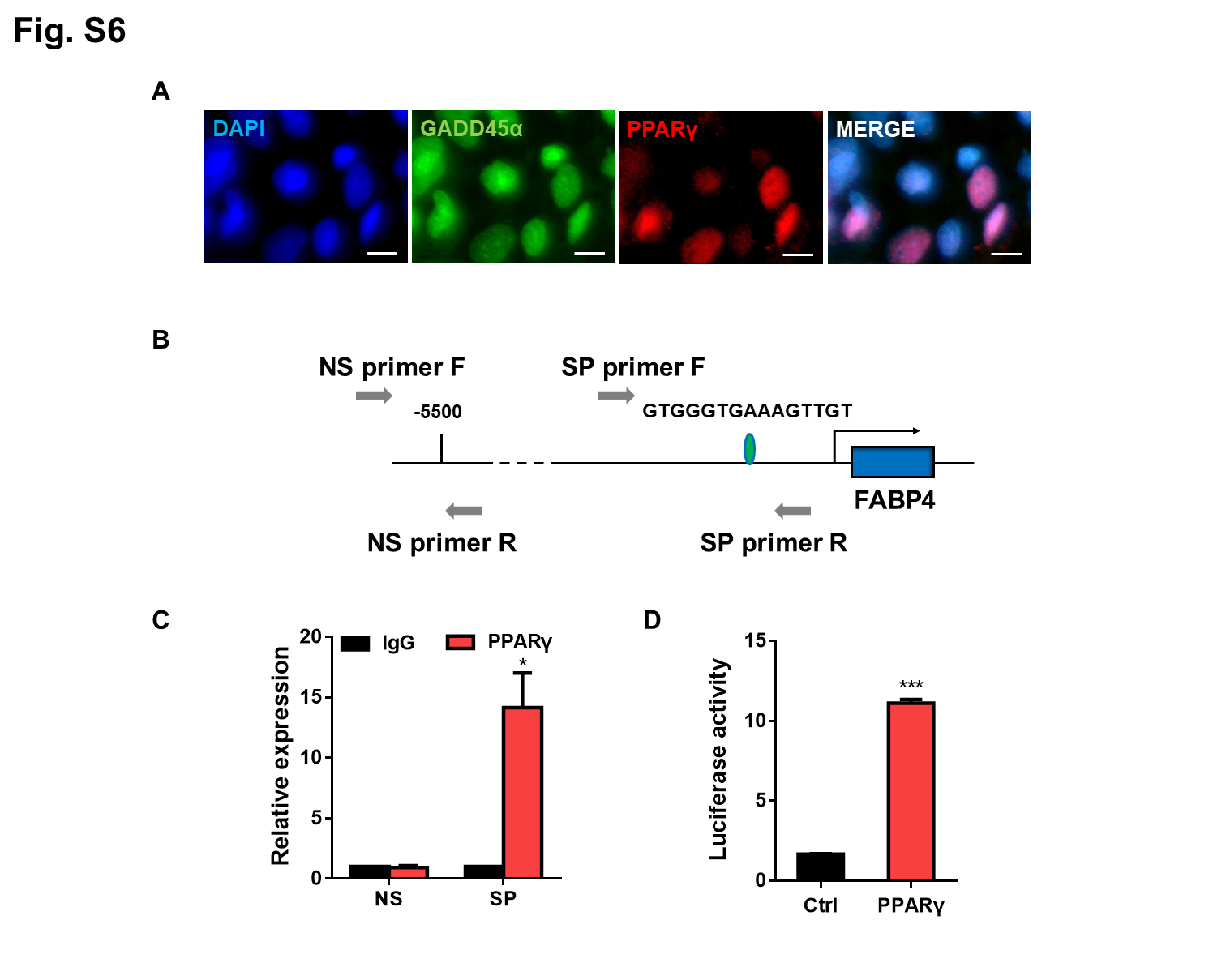

Supplement: Supplementary file 8 — Supplementary figure 6 [file 41419_2020_2802_MOESM8_ESM.tif]
